# Do Natural Experiments of Changes in Neighborhood Built Environment Impact Physical Activity and Diet? A Systematic Review

**DOI:** 10.3390/ijerph15020217

**Published:** 2018-01-26

**Authors:** Freya MacMillan, Emma S. George, Xiaoqi Feng, Dafna Merom, Andrew Bennie, Amelia Cook, Taren Sanders, Genevieve Dwyer, Bonnie Pang, Justin M. Guagliano, Gregory S. Kolt, Thomas Astell-Burt

**Affiliations:** 1 School of Science and Health, Western Sydney University, Sydney, NSW 2751, Australia; e.george@westernsydney.edu.au (E.S.G.); d.merom@westernsydney.edu.au (D.M.); a.bennie@westernsydney.edu.au (A.B.); amelia.cook@westernsydney.edu.au (A.C.); g.dwyer@westernsydney.edu.au (G.D.); b.pang@westernsydney.edu.au (B.P.); jmg221@medschl.cam.ac.uk (J.M.G.); g.kolt@westernsydney.edu.au (G.S.K.); 2 Translational Health Research Institute, Western Sydney University, Sydney, NSW 2751, Australia; 3 Population Wellbeing and Environment Research Lab (PowerLab), Faculty of Social Sciences, University of Wollongong, Wollongong, NSW 2522, Australia; xfeng@uow.edu.au (X.F.); thomasab@uow.edu.au (T.A.-B.); 4 Menzies Centre for Health Policy, University of Sydney, Sydney, NSW 2006, Australia; 5 Institute for Positive Psychology and Education, Australian Catholic University, North Sydney, NSW 2060, Australia; taren.sanders@acu.edu.au

**Keywords:** natural experiment, built environment, neighborhood, physical activity, diet, longitudinal

## Abstract

Physical activity and diet are major modifiable risk factors for chronic disease and have been shown to be associated with neighborhood built environment. Systematic review evidence from longitudinal studies on the impact of changing the built environment on physical activity and diet is currently lacking. A systematic review of natural experiments of neighborhood built environment was conducted. The aims of this systematic review were to summarize study characteristics, study quality, and impact of changes in neighborhood built environment on physical activity and diet outcomes among residents. Natural experiments of neighborhood built environment change, exploring longitudinal impacts on physical activity and/or diet in residents, were included. From five electronic databases, 2084 references were identified. A narrative synthesis was conducted, considering results in relation to study quality. Nineteen papers, reporting on 15 different exposures met inclusion criteria. Four studies included a comparison group and 11 were pre-post/longitudinal studies without a comparison group. Studies reported on the impact of redeveloping or introducing cycle and/or walking trails (*n* = 5), rail stops/lines (*n* = 4), supermarkets and farmers’ markets (*n* = 4) and park and green space (*n* = 2). Eight/15 studies reported at least one beneficial change in physical activity, diet or another associated health outcome. Due to limitations in study design and reporting, as well as the wide array of outcome measures reported, drawing conclusions to inform policy was challenging. Future research should consider a consistent approach to measure the same outcomes (e.g., using measurement methods that collect comparable physical activity and diet outcome data), to allow for pooled analyses. Additionally, including comparison groups wherever possible and ensuring high quality reporting is essential.

## 1. Introduction

The potential for city planning to promote more equitable health outcomes is of major international research and policy interest [1]. Physical activity and diet (determinants of energy balance and modifiable risk factors for chronic disease) are associated with neighborhood built environment. For example, relationships have been identified between: the presence of green space and higher levels of walking and total physical activity [2]; greater availability of supermarkets and fresh produce markets with more fruit and vegetable intake (beneficial impact), but also greater sugar-sweetened beverage intake (detrimental impact) [3]; greater use of public transport and higher physical activity [4]; and greater presence of speed limits less than 30 km/h, bicycle lanes, trees, litter, and fewer traffic calming technologies, with higher levels of cycling [5]. Whilst these studies and others have reported cross-sectional associations between built environment and physical activity or diet [6,7,8,9], evidence from longitudinal studies synthesized in systematic reviews are required to guide evidence-based policies [10]. 

Endogeneity is the mutual impact of individual characteristics and associated neighborhood characteristics on each other. For example, research suggests that neighborhood green space promotes physical activity [2] and correlations have been shown between the amount of green space and property prices [11]. Additionally, participation in physical activity is more frequent among more affluent population groups [12]. People with lower incomes tend to live in neighborhoods with less green space [2], as green space costs are capitalized into property prices. Therefore, raising house prices to make increased green space available may make these neighborhoods only accessible to healthier and wealthier people who are already more likely to be physically active.

Natural experiments are promoted as a potential answer to overcoming some challenges of endogeneity [13]. These are studies where the ‘intervention’ is occurring beyond the control and instigation of researchers. The intervention is not strictly randomly allocated, but circumstances in which it occurs are suggested to potentially help minimize the issue of endogeneity. Examples have included the ban on smoking in public places across a country on number of hospitalizations [14], reductions in neighborhood crime rates on the experience of psychological distress [15], and the provision of new local cycling infrastructure on active travel [16]. In these scenarios, circumstances change rapidly around people who tend to remain living in the same neighborhoods (although some change may happen post-intervention). Whilst acknowledging there will be some selectivity in terms of which people lived in particular areas initially, changes occurring in their neighborhood are unlikely to have been of their choice. To minimize ‘neighborhood effects’, tracking the impacts of interventions within residentially stable populations is suggested [17].

Previous systematic reviews have tended to place less emphasis on endogeneity, including studies of varying design [6,18,19,20]. In the current study, the aim is to review studies focusing specifically on natural experiments of the built environment in neighborhoods (referred to hereafter as the ‘exposure’), occurring around residentially stable populations, which have measured changes in physical activity or diet. Study characteristics, study quality, and impact of exposures on physical activity and diet are summarized. The review aims to answer the following questions in relation to natural experiments of neighborhood built environment:

- What were the characteristics of studies, including exposure type (e.g., food retail, green space), study design, follow-up duration, recruitment strategies, retention level, study aims and outcome measures?
- What was the quality level of included studies based on assessment of risk of bias?
- What was the impact of exposures on physical activity and diet of residents?

## 2. Materials and Methods 

Preferred Reporting Items for Systematic Reviews and Meta-Analyses (PRISMA) guidelines were followed throughout this review [21]. 

### 2.1. Search Strategy

The following databases were searched: Embase (OVID); MEDLINE (OVID); PubMed; Web of Science; and CINAHL. All authors reviewed the search strategy and the lead author carried out the search. Keywords relating to study design, the built environment, health and health-related behaviors were used (apart from in the CINAHL search, which excluded a study design keyword—the number of returned titles was low with inclusion of study design terms). Details of the full search strategy for the Web of Knowledge database are provided in Table S1. Similar keywords relevant to search other databases and MeSH headings were used where available. Searches were conducted in May 2014. An updated search was run in May 2017. Secondary searches of reference lists of included articles and relevant reviews were also conducted to identify eligible studies. 

### 2.2. Study Eligibility

Eligibility criteria for this review aligned with the “Population”, “Intervention”, “Comparisons”, “Outcomes”, “Study designs” (PICOS) strategy [22] as follows:

- Population: Studies included any age, gender, and characteristics of the population/target site. Participants needed to be reported in papers to reside and be residentially stable in the neighborhood where the exposure/s occurred (i.e., participants resided in the same neighborhood for the duration of the study—samples included the same participants at baseline and follow-up).
- Intervention/exposure: A change in the local environment was defined as a development in existing (regeneration) or introduction of new public built infrastructure to the area in close locality to where individuals reside (e.g., their neighborhood that could potentially impact on physical activity or diet, such as the introduction/regeneration of supermarkets or local food markets, rail lines, green space and cycle routes).
- Comparisons: Studies were included if the impact of an exposure was assessed based on changes in outcomes over time (i.e., pre-post exposure) in the same sample of participants, or changes in these outcomes over time in a comparator group that did not receive the exposure.
- Outcomes: Studies were included if they measured physical activity or diet (no restriction on the measurement method). Studies including a direct proxy of behavior were included (e.g., usage of a facility for cycling or walking).
- Study designs: Studies were included if they were reported to be, or appeared from reading, natural experiments (built environment change not instigated by researchers).

Peer-reviewed articles published in English were included. No limitation on year of publication or length of follow-up was set.

### 2.3. Exclusions

Studies were excluded if: (i) they were reported as comprising multi-component exposures (e.g., exposures which explicitly reported social interventions, including promotional marketing to encourage use of built environment features in addition to infrastructure change), as it would not be possible to attribute changes in physical activity and diet specifically to the built environment change; (ii) changes were internal housing improvement (e.g., heating/electrical improvements in housing); (iii) no clearly defined or measured exposure was studied (e.g., studies which compared groups exposed to different, or pre-existing built environments but with no specific change to the built environments within groups); (iv) changes were stated explicitly to occur outside of residential neighborhoods (e.g., workplace or public transport developments); (v) they explored the impact of a detrimental change in built environment (e.g., natural disaster or demolition); (vi) no physical activity or diet outcomes were reported (e.g., focus upon self-sufficiency or criminal behavior); and (vii) participants were not residentially stable (e.g., participants were reported as residents recruited from the same area/neighborhood at baseline and follow-up/s but the sample was not reported to consist of the exact same cohort at baseline and follow-ups).

### 2.4. Data Extraction and Appraisal

Review instructions were developed by the lead author (Freya MacMillan) and followed by all authors to ensure consistency in article screening, data extraction and risk of bias ratings. Standard data extraction datasheets were utilized. Eight researchers (Amelia Cook, Andrew Bennie, Bonnie Pang, Fran Moran (see Acknowledgements), Genevieve Dwyer, Dafna Merom, Taren Sanders, Brendon Hyndman (see Acknowledgements)) reviewed a selection of titles and abstracts. Two researchers (Emma S. George, Freya MacMillan) who were not involved in the initial screening independently screened ten percent of identified references deemed ineligible based on titles and/or abstract. A further two researchers not involved in initial screening (Thomas Astell-Burt, Xiaoqi Feng), screened all references deemed eligible based on title and abstract review. Three researchers (Freya MacMillan, Emma S. George, Justin M. Guagliano) reviewed full-text articles and undertook initial data extraction. Seven independent researchers (Thomas Astell-Burt, Andrew Bennie, Amelia Cook, Bonnie Pang, Gregory S. Kolt, Taren Sanders, Xiaoqi Feng) reviewed a sub-set of full-texts and extracted mean data. 

A tool for assessing methodological risk of bias in natural experiments exploring the impact of built environment change on physical activity exists [23]. As the rigidity of this tool has been questioned for this type of research [24], a more pragmatic set of items were used in this review. Included studies were assessed using a 9-item tool including two items from the Cochrane Collaboration for assessing risk of bias tool on attrition (incomplete outcome data) and reporting bias (selective outcome reporting) [25]. Seven items considering bias due to study design, sampling approach, confounding and adjustment, outcome measurement objectivity, power and attrition rate effect on power, levels of exposure, and exposure use/adoption were developed based on important considerations for natural experiments discussed in the UK. Medical Research Council (MRC) guidelines [26,27] (see Table 1). 

### 2.5. Synthesis of Results

Table 2 summarizes included study details. Analytic items used to organize the extracted data were: authors, year of publication and study location; study aims; target population descriptive characteristics, recruitment methods and study duration; study design, including development description; outcome measures (studies had to have a measure of physical activity or diet to be included but all other lifestyle and health outcome data were extracted—the results section lists all measures identified) and methods; and results for full sample and any sub-group analyses. 

Four researchers (Emma S. George, Freya MacMillan, Genevieve Dwyer and Fran Moran (see Acknowledgements)) rated studies (present or not present/unclear) based on what was reported in each article for the potential sources of bias (detailed above). Studies were scored out of 9 (one mark for each item). When five independent reviewers (Gregory S. Kolt, Dafna Merom, Xiaoqi Feng, Thomas Astell-Burt and Andrew Page (see Acknowledgements)) conducted risk of bias ratings, the agreement rate was 95.8%. Reviewers discussed discrepancies throughout the review process until consensus was achieved. The analytical approach taken was a narrative description (meta-analysis was considered but rejected due to large heterogeneity in reported outcomes (see Results section)).

## 3. Results

**Figure 1:**
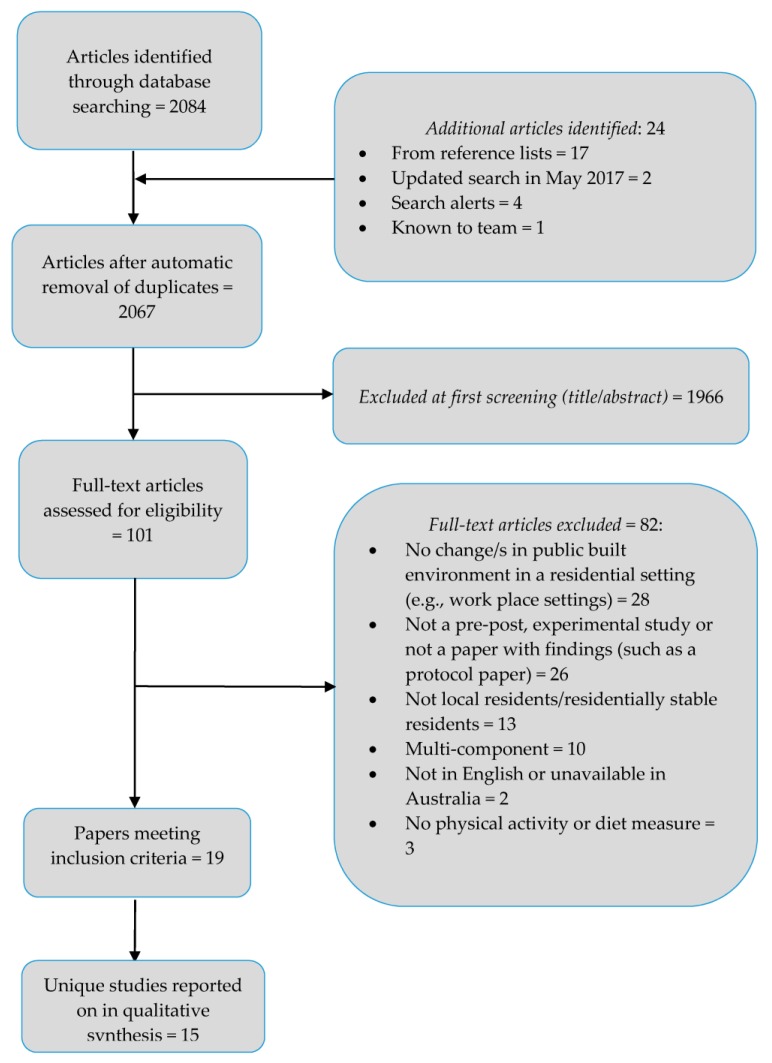
Flow diagram of study selection.

A total of 2084 references were identified from initial database searching, plus 24 references from other sources (e.g., identified from reference lists and search alerts, Figure 1). Following removal of duplicates and exclusion of 1966 references based on an initial title and abstract screening phase, 101 references were included for further review. Reasons for excluding studies at the final screening stage are detailed in Figure 1 (further detail is provided in Table S2), with the most common reasons being that the studies did not meet inclusion criteria relating to built environmental change (*n* = 28) or due to study design (e.g., no results published, such as in a protocol paper, *n* = 26). The remainder of this results section focuses on the 19 eligible papers identified for this review.

### 3.1. Study Characteristics

Nineteen papers, reporting on 15 different exposures employing longitudinal natural experimental designs were identified and included in this review. Throughout the results/discussion section, the 15 unique experiments are considered (the number of papers that are associated with the specific experiment are referenced). Five studies focused on cycle and/or walking trails [29,35,36,37,38,40], four on rail stops/lines [33,34,39,41,42], two on park and green space [28,43], and four on food retail (including supermarkets [30,31,32,45,46]) and farmers’ markets [44]). Two papers reported on the same supermarket developments (one in Glasgow, Scotland [30,31], and one in Leeds, England [45,46]), two reported on the same cycle/walk trail exposure [37,38] and two reported on the same rail stop introduction [41,42]; each paper addressed different health outcomes or extending analyses. The publication date ranged from 2002 [45] to 2016 (*n* = 2 studies) [34,40]. Ten studies were conducted in the U.S. [29,32,33,34,35,36,39,41,42,43,44], three in the UK [30,31,37,38,45,46], one was conducted in South America [40] and one was conducted in New Zealand [28].

### 3.2. Study Design and Follow-Up Duration

Eleven/15 studies (73%) were of a single group pre-post/longitudinal design [33,34,35,36,37,38,39,40,41,42,43,44,45,46], while the remaining four studies included a comparison group [28,29,30,31,32]. Two studies had more than one follow-up data collection time point at one and five [35] months and at 12 and 24 months [37,38]. 

Follow-up duration ranged from two months [36,44] to 36 months [40] after exposure (duration of ≤6 months, *n* = 6 studies [28,32,34,35,36,44]; 6.1–12 months, *n* = 6 studies [30,31,33,39,41,42,43,45,46] and >12 months, *n* = 2 studies [37,38,40]). One study collected follow-up data between 2 and 12 months following exposure [29].

### 3.3. Recruitment Procedures and Retention

Of studies that reported on data collection and recruitment methods, a variety of approaches were utilized including: door-to-door visits in two studies [41,42,45,46], door-to-door visits at baseline followed by telephone or mail at follow-up in one study [44], mail notification of the study followed by a door-to-door visit in one study [33], solely via mail in four studies [30,31,34,37,38,43], flyer drop-off to doors in one study [29], mail notification followed by telephone data collection in one study [36], solely via telephone in three studies [32,39,40] and via schools using information letters for students and parents in one study [28]. Representativeness of the sample, based on descriptive census or other local and national data, was reported in four studies [36,37,38,44,45,46]. In studies reporting on the number of individuals invited to participate, the study invitation acceptance rate ranged from ~1% to 15% in three studies [30,31,34,37,38], 31–47% in six studies [32,33,35,36,43,45,46] and above 90% in two studies [40,44], with studies using only mail or flyer drop-off recruitment showing the lowest acceptance rates. Incentives, to support recruitment and retention, were reported in four studies [28,30,31,34,45,46].

Total sample size at baseline (regardless of the number of groups) ranged from 92 [44] in a study exploring the introduction of farm stands, to 3516 [37] in a study on the impact of a cycle/walk route. At final follow-up, total sample size ranged from 47 [33] in a rail stop study to 1510 [37] in a cycle/walk route study. Including all studies, the median sample size at baseline was 603, with eight studies reporting a sample size ≤the median [28,29,30,31,33,34,35,43,44]. 

Participant retention from baseline to final follow-up ranged from 45% [32,43] to 84% [28]. Two studies provided a sample size calculation [28,40] and a further two studies reported a target sample size [44,45,46]—the remaining studies did not report either. Only one study targeted children [28]. Three studies reported specifically targeting recruitment from socially deprived/low-income areas [30,31,44,45,46]. 

### 3.4. Aims and Outcome Measures

The reported primary aim of studies varied considerably, as did the methods of assessment. Most studies (11/15) included physical activity as a primary outcome [28,29,34,35,36,37,38,39,40,41,42,43]. Five studies included objective measures of physical activity—two used accelerometers only [28,33], and three utilized accelerometers combined with GPS [29,34,41,42]. One study used a physical activity diary [35] and five included self-report surveys [36,37,38,39,40,43]. The remaining four studies focused primarily on diet [30,31,32,44,45,46]. Of those studies assessing dietary intake, one study measured food consumption with diaries [45,46], and three reported fruit and vegetable consumption using questionnaires of usual consumption: per day [30,31] over the past week [44], or consumption of specific fruits and vegetables over the previous month [32]. Additional outcome measures reported in studies were BMI, obesity and health (including psychological or mental health outcomes, collectively termed hereafter as well-being). BMI was measured by a health professional or researcher in two studies [28,41,42] and relied on self-reported weight and height in three studies [32,36,39]. Of these studies, one also included self-reported obesity [39]. Self-reported health and well-being was measured in two studies using the General Health questionnaire [30,31,36]. 

### 3.5. Study Quality

Reporting varied considerably across studies, with 13/15 studies having a high-risk score in ≥4/9 of the risk of bias items (Table 3). As previously mentioned, only 4/15 studies included a comparison that did not receive the exposure under study [28,29,30,31,32]. Level of exposure was explored in 5/15 studies based on distance to the exposure [34,37,38,40,43,45,46], whilst 6/15 studies examined outcomes based on use/adoption of the exposure [30,31,32,36,39,41,42,45,46]. Sample representativeness, by comparison to census or other local population data, was included in 8/15 studies [30,31,33,35,36,37,38,40,44,45,46]. 

Although studies reported on all outcomes stated in the aims/methods, descriptive (e.g., mean/median) or statistics (e.g., *p*-values) were missing at pre/post time-points in 7/15 studies [30,31,32,33,37,39,43,45,46]. Incomplete data was addressed by use of data replacement and/or sensitivity analyses in 6/15 studies [29,32,37,38,39,40,41]. 

Differences in participant characteristics (between baseline and follow-up or between exposure and comparison groups at baseline) were reported and/or adjusted for in 11/15 studies [28,29,30,31,32,34,36,37,38,39,40,41,45,46]. The majority of studies relied on participant self-report data, with only 5/15 studies including an objective measure of physical activity [28,29,33,34,41,42] and researchers/health professionals measuring BMI in 2/15 studies [28,41]. Power calculations or target sample sizes were mentioned for 6/15 studies [28,32,38,40,41,45,46], but details of calculations were only stated in two papers [28,40].

### 3.6. Impact on Outcomes

#### 3.6.1. Findings from Controlled Studies 

Of the four studies [28,29,30,31,32] that included a comparison group (Table 2), one study reported improvements in self-reported fruit and vegetable intake 12 months after a new supermarket was introduced, but improvements were found both in the environmental change and comparison groups [30,31] (high risk in 5/9 risk of bias items). In this same study, a slight beneficial impact on well-being was found in the experimental group [30,31]: at 12-month the prevalence of poor psychological health had significantly decreased by 31% compared to the comparison group (only a 3% decrease). No changes over time or between groups were reported on health/behavior outcomes in the full samples of two controlled studies, one of which also introduced a supermarket [32] but had lower total risk of bias score (high risk in 4/9 items) and another introducing green space [28] (high risk in 4/9 items). In another study [29], residing in areas where bicycle routes were introduced was negatively correlated with bike trips and minutes cycling (high risk in 4/9 items).

Significant changes in sub-groups of controlled studies are reported in Table 2. Two studies, [30,31,32] conducted sub-analyses based on use/adoption of supermarket exposures. One of these studies reported reduced odds of poor psychological and physical health (see table for odds ratios) in those adopting the new supermarket over those that did not [30,31]. The other study did not report any changes in sub-groups [32].

#### 3.6.2. Pre-Post Study Findings 

Findings from pre-post evaluation studies (Table 2) indicated significant improvements in at least one outcome for the total sample in 4/11 uncontrolled studies, for which high risk of bias ratings were given in 3/9 [40] and ≥7/9 items [35,43,44]. Of these studies, the one study rating lowest risk of bias overall found that a walking/cycling route increased leisure time walking by 14 min per week after 36 months in Brazil [40]. 

No changes were reported in total samples of 4/11 uncontrolled studies, which measured physical activity changes after introductions of rail stops [33,34], a cycle path [37,38] or diet after a supermarket introduction [45,46]. One study reported detrimental impacts on vigorous physical activity eight weeks after the introduction of a multi-use trail [36]. Two studies did not measure changes in their overall sample [39,41,42].

Nine out of 11 uncontrolled pre-post studies analyzed data based on exposure level, where level was defined based on the use and/or adoption of the exposure (*n* = 4) [36,39,41,42,45,46] and/or home distance to the exposure (*n* = 5) [34,37,38,40,43,45,46]. Five of these nine studies reported significant beneficial changes in sub-groups based on expected hypotheses, one of which had a high risk of bias in 8/9 items [43] and the remainder of studies in ≤4/9 items [37,38,40,41,42,45,46]. For example, a new walk/cycle route increased total physical activity on average by 12.5 min/week per km closer to the exposure [38]. Two studies reported unfavorable changes [34,36], with a 77% reduction in vigorous physical activity minutes/week following introduction of a multi-use trail in those that had ever used the trail [36] (high risk of bias in 5/9 items). In comparison to those not using the trail, those who ever used the trail were less likely from baseline to follow-up to have increased walking by 30 min/week and 45 min/week [36]. The remaining study reported no significant changes [39]. 

## 4. Discussion

### 4.1. Summary of Overall Findings of This Review 

This paper systematically reviewed natural experiments of the built environment occurring around residentially stable populations to report on study characteristics, study quality, and impact of changing built environment on physical activity and diet. Limited evidence was found to support built environment as an important factor influencing these outcomes, with large variation in results (8/15 studies reported at least one beneficial impact on these behaviors/health). However, study design (lack of a comparison group), underpowered sample sizes, the use of a wide array of outcome measures and limited reporting in some included studies, have made it challenging to draw overall conclusions in this review. 

One of the higher quality studies (5/9 risk of bias items rated high risk) with a comparison group reported a small beneficial impact on well-being following the opening of a supermarket [30,31]. In 6/10 studies including sub-group analyses based on exposure level and use/adoption, these studies found improvements related to well-being, physical activity, BMI, and fruit and vegetable intake in those using and adopting the exposure, although sample sizes were small. These findings suggest that there is potential to improve health and behaviors by improving the built environment. However, to accurately inform policy, there is a need for future studies in this area to closely follow guidelines on conducting and fully reporting on natural experiments [26,27]. A discussion of how our findings support and add to this previously published guidance follows, with each area of the review covered in detail as per the aims stated in the Introduction.

### 4.2. Study Characteristics

#### 4.2.1. Design

Natural experiments, by definition, are not designed by researchers and rarely, if ever, are designed with a specific aim of improving physical activity and diet—the impact of the natural experiment on such behaviors is most often a by-product of the exposure. Challenges for researchers in such experiments are therefore: defining causal pathways (e.g., the impact of the exposure on a range of outcomes); selecting appropriate outcomes to assess potential impacts on health; and identifying robust methods to measure changes in outcomes. The U.K. Medical Research Council (MRC) recommendations on conducting natural experiments [26,27] provide comprehensive best-practice guidance on identifying when natural experiments are appropriate, the methodological and analytical considerations in regards to reducing bias, and effective reporting. Two/six studies in the current review, published after the introduction of the MRC guidelines, cited their work, implying that this resource is not reaching or being implemented in practice. Implementation issues could be due to the inherent nature and challenges associated with this type of research, often outside the researcher’s control (e.g., timeline and budget); however other issues could and should always be addressed (e.g., consideration of confounders in analyses). Few included studies referred to their study as a natural experiment, and as such, lack of recognition for the relevance of the guidelines may also have influenced and limited their use in previous studies. 

#### 4.2.2. Outcomes, Recruitment and Retention

The range of health outcomes explored across studies was limited. As a result, important changes in outcomes may have been missed due to the lack of measurement or the use of inappropriate tools. Small, yet beneficial, changes in behaviors that shift people from not meeting to achieving or exceeding public health recommendations can have important health impacts [47]. Although physical activity and diet were measured in all studies, no studies reported on these behaviors in relation to meeting public health recommendations (e.g., % population achieving guidelines). Proxy measures of health and behaviors are useful (e.g., awareness and use of a fresh food store as an indicator for diet) to identify if and to what extent the infrastructure is known and used. However, used alone as a single measure, these may not provide enough evidence to draw conclusions on true impact (e.g., a park environmental change could increase both physical activity (beneficial impact), or sedentary behavior (detrimental impact)). None of the included studies incorporated a health economic analysis—a recommendation for natural experimental research [26,27]. This is likely due to the fact that natural experiments are not undertaken for the primary purpose of improving health and so data to inform cost-effectiveness may not have been considered from commencement of built environment changes. 

Recruitment and retention is challenging in natural experiments. To reach target sample sizes, recruitment needs to be well planned and, although based on the findings of this review, incorporating face-to-face contact appears important, this is likely not feasible in large population studies. The use of incentives may result in the recruitment of biased samples; however, offering incentives may assist in overcoming the difficulties associated with recruiting disadvantaged populations (e.g., individuals from low socio-economic status (SES) backgrounds) [48] and should thus be considered. Considering use of incentives in future is particularly important as a recent review suggests that the most socioeconomically advantaged groups may benefit the most from physical activity and active transport built environment improvements [20], which may be because they are more likely to participate in this type of research than the most socioeconomically disadvantaged. In addition to recruitment, retention is an issue, particularly in areas where residential in- and out-migration is high. Systematic reviews of the use of incentives for retention in cohort [49] and RCT [50] studies support their use. Incentives did not appear to reduce attrition in the current review, however only four studies reported using them. Routinely collected data (as is recommended for evaluation of natural experiments [26,27]) was not used in any of the included studies and should be explored as a way of collecting data whilst avoiding recruitment/retention issues in future studies.

#### 4.2.3. Geographic Location of Studies and Study Duration

Similar to previous reviews on the association of built environment and health [7,51], short follow-up duration, limited number of follow-up data points, and the clustering of studies in primarily high-income countries (mostly the U.S. and UK) was evident, with a small number of studies specifically recruiting from low SES areas. Timing and duration of data collection is important. Due to timeline changes, baseline data collection in one study included in this review [32] occurred three years before the exposure. The impact of such delays in timeline, although out of the control of the researcher, need to be considered and flexibility in data collection is needed to adjust for changes in the timing of the development of planned infrastructure. Another study in this review [29] had a variation of 2–12 months in follow up timing, resulting in some participants having more time than others to adopt change. Changes in intermediary health or behavior outcomes may be evident first before long-term outcomes. Depending on the outcome, impacts may be expected to occur close to or long after the exposure and can be short, or longer-lived. Logic modeling [52] is recommended to help identify what and when particular outcomes should be incorporated into evaluation, to assist with amendments required to data collection plans, and to consider sources of bias and ways of minimizing impact on findings [26,27]. Adequate level of exposure for meaningful differences in outcomes to occur should be considered as well as analyzing changes in built environment and resultant effects across several areas (SES and geographically diverse). The impact of built environment change may differ in middle and low-income countries in economic transition and increased urbanization, and this needs to be taken considered. Previous research shows that built environment modification did not achieve intended outcomes on the total target group exposed, but when stratified by SES [53], or migrant status [54], developments were found to minimize gaps in health inequality. The social distribution of impacts of environmental change should be considered in future research.

### 4.3. Study Quality

Comparison groups are important to provide less biased, or more precise estimates, of the impact of changes in built environment on changes in outcomes. Only four studies included a non-exposure comparison [28,29,30,31,32] and ten studies compared sub-groups with different levels of exposure based on distance [37,38,40,43,45,46] or use/adoption of the exposure [30,31,32,36,39,41,42,45,46]. Sub-analyses based on use/adoption provide a more accurate reflection of true impact of exposure—in studies only reporting on total sample results, the effects of exposure on behavior and health are blurred (from mixing data of those that use the exposure with those that do not use the exposure). Researchers should consider innovative ways of reliably capturing use/adoption, such as utilizing smart phones for real-time spatial tracking [55] or automated attendance recording [56]. To clearly identify the impact that built environment can have on physical activity and diet outcomes, pooled data from studies that have split their analyses based on use/adoption of the exposure with large enough samples to detect effects are required. Although distance from exposure might be an indicator of awareness and use, this is a proxy measure and direct measures may be more accurate and meaningful. A framework for considering exposure in natural experiments has been published [24]. Understanding adoption and long-term use is vital in order to maximize use of built environments. Longitudinal qualitative research is recommended for this purpose, such as that planned in the protocol paper [57] for a study included in this review [37,38].

It is often impossible to find comparison groups in natural experiments that have a change in exposure acting in the opposite direction (e.g., a community that receives a new supermarket versus another community that is similar in almost all aspects but has a supermarket removed), in a similar community (e.g., same SES background). Detailed descriptions of built environment changes are essential in this type of research and studies need to consider the potential impact of other significant changes in built infrastructure, other than the exposure under investigation, on outcomes. Ideally, these other changes should be measured using an objective measure, for example using time-varying analyses as a measure of confounding [58]. 

### 4.4. Strengths and Limitations 

The MRC guidelines recognize the complexity of systematically reviewing natural experimental literature [26,27]. A rigorous and systematic approach was taken in this review; however, the findings of this review should be considered in light of potential limitations. Risk of bias items used in this review included validated items and newly developed non-validated items appropriate for natural experiments, which reflected key biases highlighted in natural experiment guidelines [26,27]. Using this tool allowed discussion of results in the context of study reporting quality and similar to a recently published systematic review of built environment exposures on physical activity and active transport [20], identified limitations in study quality across included studies that should be considered when designing future evaluations. Also, similar to this recently published review [20], we excluded studies that reported enhancing environmental changes with other components, including local awareness campaigns, to examine the impact of built neighborhood environment changes alone. It is possible that some studies including such components in addition to built environment changes were included due to omitting such components from their reporting. It is acknowledged that multi-component interventions result in the most impactful health behavior change programs [53,59,60]. The aim of this review was to restrict inclusion to those studies only assessing the impact of changes to public neighborhood built environment features. Had multi-component studies been included, it would not be possible to determine changes attributed to the built environment elements on lifestyle behaviors. Studies were included if it was stated that participants were neighborhood residents regardless of if a definition was reported or not. Only peer-reviewed articles were included in this review to ensure a level of quality—important findings may have been missed by omission of grey literature. Individual authors were not contacted for additional study information and thus it was not clear for several risk of bias items if measures were not in place to minimize bias, or were not reported on. Some studies may have been rated as having a high risk of bias due to limited reporting in associated articles rather than actual study design and conduction. The challenges of summarizing findings across natural experiments were particularly noted in this review, as was the case in a similar review specifically of physical activity and active transport built environment interventions [20], due to the vast and varying ways of reporting on the same outcome (e.g., for physical activity—percentage residents using a park, total time spent walking, bouts of activity) and quality of reporting. Although estimation of effect size across studies was not possible for this reason, the findings of this review can be used to inform research priorities in future and provides a qualitative interpretation summarizing current evidence on the impact of built environment on physical activity and diet from natural experiments.

## 5. Conclusions

Identifying the impact of built environment change alone on physical activity and diet outcomes is important for establishing the level of focus and investment that should be made on built environment in socio-ecological interventions. The quality of evidence published to date, including natural experiments, is scarce and limited. It would be surprising if the built environment were not an important standalone driver of physical activity and diet, but available evidence in the research literature thus far is not strong enough to lead to a definitive conclusion. Further research is needed to develop a consistent approach to measure the same outcomes (e.g., consensus for how to measure and report physical activity in these types of studies), so that pooled meta-analyses can be conducted.

Taking into consideration the differences in design and reporting, the findings of this review cannot definitively support nor rule out the existing belief among urban planners and policy makers, that changes to the built environment are powerful interventions not only for preventive health and well-being, but also for improving physical activity and diet outcomes at the community-level. The interventions in this review were largely ineffective and therefore such approaches require further testing. It may be that the impact of changing built environment on health outcomes and physical activity and diet is small, however, if the change affects large population numbers, even small changes in behaviors will have the potential to reduce disease risk and prevalence at a population level [61]. The findings of this review are useful for researchers and policy makers to assist in effectively planning longitudinal evaluations of natural experiments involving built environment changes. 

## Figures and Tables

**Table 1:** Risk of bias item descriptions.

Risk of Bias Item	Label	Description
1	Study design	Did the study include a comparison that did not receive a change in built environment?
2	Sampling approach	Did the sampling approach generate a sample that reflected the wider population of interest (e.g., reporting that there were similar characteristics in the sample in comparison to census/other population data for the area of interest)?
3	Incomplete outcome data (assessments were made for each outcome/class of outcomes)	Were incomplete outcome data adequately addressed? (e.g., were details of how missing data were handled reported, such as ITT analysis? Was sensitivity analysis conducted, with *n* = reported for outcomes at all time points?)
4	Selective outcome reporting	Are reports of the study free of suggestion of selective outcome reporting? (e.g., all outcomes mentioned in methods are reported on in the results section for all groups/time points)
5	Adjustment for differences in sample characteristics	Were characteristics of sites similar at baseline? If confounders were identified, were they appropriately adjusted for in analyses? In longitudinal studies without a comparison, were characteristics of the follow-up sample similar to the baseline sample or were confounders adjusted for in analyses?
6	Outcome measurement objectivity	Was an objective assessment of health outcomes/behavior included (a measure free from participant subjectivity was used)?
7	Reporting of power calculation and attrition rate effect on power	Was a power calculation reported and the study was adequately powered to detect hypothesized relationships?
8	Levels of exposure	Was an analysis undertaken exploring changes in outcomes based on different levels of exposure (e.g., based on distance to the exposure)?
9	Exposure use	Was an analysis undertaken exploring changes in outcomes based on use/adoption of the exposure?

**Table 2:** Summary of included study characteristics and longitudinal findings.

Ref., Country, Exposure	Aim	Recruitment Process, Study Population, and Data Collection Time Points	Study Design, Exposure Details and Comparison	Outcome Measures	Efficacy on Outcomes (only Significant Changes in Longitudinal Findings Reported)
**Controlled studies**					
**Green space**					
Quigg, R., et al. (2012) [28]. Dunedin, New Zealand Exposure: Green space	To assess whether an upgrade of playgrounds in a neighborhood was associated with changes in local children’s physical activity levels.	Recruitment: Local authority community boundaries used to identify the intervention neighborhood. Six intervention and four comparison community schools were invited to participate via information letter to students and parents (*n* = 4 intervention and *n* = 4 comparison schools accepted). All children aged 5–10 years in kindergarten to grade 4 residing in the community were given information and parent and child consent forms to take home. A sample size calculation was used (*n* = 100 in each group was required). *Incentive*: Family swim vouchers worth $8 were provided as an incentive to wear accelerometers at each time point. A swimming bag worth $3.50 and goggles and a Frisbee worth $8 were given as incentives for completing the survey at T1 and T2. *Participants*: T1 = *n* = 184. T2 = *n* = 156 (15% loss to follow-up: 10%, *n* = 9, from the comparison group and 20%, *n* = 19, from the intervention group). Participants with at least 1 day of accelerometer wear at T1 and T2 = *n* = 138 (*n* = 132 had 4 or more days). Survey data collection rate was 93% (128/138) at T1 and 96% (133/138) at T2. *Time points*: T1 = October–December 2007. T2 (1-year post-T1 and 3-months post park upgrade) = October–December 2008 (spring).	Study design: Pre-post study with control. *Exposure*: playground upgrades in 2/6 existing parks in the intervention community. One playground received 10 new components including: play equipment; seating; additional safety surfacing and waste facilities installed; and the removal of two existing components. The other playground received two new play pieces and modification to an existing piece of equipment. *Comparison*: a similar matched community not undergoing park regeneration. Interactions between BMI-Z score and group on physical activity explored.	Total daily physical activity (accelerometer) BMI Z-score (researcher measured)	Change in total daily physical activity was associated with an interaction between BMI and the participant’s community of residence (*p* = 0.006), with the intervention being associated with higher levels of PA for children with lower BMIs, but lower levels of PA for children with higher BMIs. Participants in the intervention group, compared to the comparison group, had increases in total PA for those with BMI z-scores below 0.4 and lower total PA for those with BMI z-scores above 0.4. No other significant changes were reported.
**Bike/walk trails**					
Dill, J. at al. (2014) [29]. Portland, Oregon, United States. Data from the Family Activity Study (FAS) Exposure: bike/walk trails	To evaluate changes in physical activity and active transportation associated with installation of new bicycle boulevards.	Recruitment: Participants resided in street segments scheduled for bicycle boulevard installation (0.9 to 4.2 miles long). Residents living on and within 1000 ft of the selected street segments were recruited via door delivered flyers, of accessible housing units, and mailed to residents in inaccessible housing units (*n* = 54,381). The comparison consisted of 11 control street segments (1.0 to 5.7 miles long), similar in urban form and demographic characteristics (especially in terms of bicycle infrastructure). 3.1% of the estimated eligible population was recruited at T1. Participants: T1 = baseline sample, *N* = 490; *n* = 307 adults in the exposure group and *n* = 183 in the comparison group. Time points: T1 = 2010–2011 and T2 = 2012–2013. Follow-up varied between 2 and 12 months after exposure.	Study design: Pre-post with control. Exposure: Bicycle boulevard installations across multiple areas. Comparison: No bicycle boulevard installations introduced.	Physical activity; MVPA, number of bike/walk trips; number of minutes walking/cycling (accelerometer combined with GPS)	Bicycle boulevard introduction was negatively correlated with bicycling (if >10 min, *p* = 0.00) and the number of bike trips (if >0, *p* = 0.06).
**Supermarkets**					
Cummins, S., et al. (2008) [30].^a^ Cummins, S., et al. (2005) [31]. ^b^ Glasgow, UK Exposure: supermarket	To examine the impact of a new food retail development on diet and health and well-being. To determine the effect of the introduction of a hypermarket in a deprived community on fruit and vegetable consumption and health including psychological health.	Recruitment: Study site boundaries were identified using postcode districts of areas with the main shopping facilities. Random sample of households surrounding two sites identified from a postcode address file (within 1 km). Postal questionnaires sent to homes (*n* = 3975). Postal reminders sent after 2 weeks and a 2nd reminder after another 2 weeks (including the survey again), to those that did not reply. Control and intervention response rates were 15.50% and 14.84%, respectively, at T1 and 71.29% and 65.18% at T2. Incentive: At follow-up £10 shopping vouchers, not for the exposure store, were given to survey responders. Participants: Surveys were completed by 603 participants at T1 (15.16% response rate) and 412 participants at T2 (68.40%). At T1 293 participants were in the intervention group and 310 in the comparison group.	Study design: pre-post with control. Exposure: Building of a new hypermarket. Comparison: a deprived comparison area not undergoing significant infrastructure change. For sub-analyses: those that switched to using the new supermarket compared to non-switchers.	General and psychological health (Self-rated health and well-being survey) Diet—fruit and vegetable consumption (self-report survey)	An improvement in poor psychological health was found (−12.13%, *p* = 0.017) in the intervention group from T1 to T2. Vegetable (*p* = 0.01), and fruit and vegetable combined (*p* = 0.003) consumption improved in the comparison group from T1 to T2. Following adjustment for baseline psychological health, the odds of poor psychological health was reduced (OR 0.42, 95% CI 0.19 to 0.92) in switchers compared to non-switchers. Further adjustment for other confounders further reduced the odds of poor psychological health in switchers compared to non-switchers (OR 0.24, 95% CI 0.09 to 0.66). Adjusted odds of having poor health increased in the intervention group compared to the comparison group (OR 1.52, 95% CI 0.77 to 2.99).
		At T2 191 participants were in the intervention group and 221 in the comparison group. A sub-analysis was conducted comparing data of those that switched their food purchasing to the new hypermarket (switchers in the 2005 paper: *n* = 66; switchers in the 2008 paper (*n* = 61; *n* = 58 from the intervention group and *n* = 3 from the comparison group) compared to those that did not. Time points: Hypermarket opening = November 2001. Survey T1 = October 2001. Survey T2 = 1 year post-baseline and 10 months post supermarket opening.			Unadjusted odds of poor health improved in switchers (OR 0.62, 95% CI 0.34 to 1.11). No other significant changes were reported.
Cummins, S., et al. (2014) [32]. Philadelphia, PA, USA Exposure: supermarket	To determine the effects of the opening of a new supermarket, in a community considered a food desert, on BMI, daily fruit and vegetable intake and perceptions of food accessibility.	Recruitment: Adult residents living within 1.5 miles of the supermarkets from two neighborhoods randomly selected from a directory list and using random digit dialing. Incentive: Respondents were given $20 for participation. Participants: Overall response rate was 47.2% at T1 = *n* = 1440 (response rate of 47.4% in the intervention group, *n* = 723 and 47.0% in the comparison group, *n* = 717). The response rate was 45.5% at T2 = *n* = 656 (response rate of 43.7% in the intervention group, *n* = 311 and 43.7% in the comparison group, *n* = 48.9%). Time points: The supermarket opened in December 2009. T1 (June–September 2006). T2 (June–November 2010, at least 6-months post-intervention).	Study design: Pre-post with control. Exposure: Opening of a new supermarket in a food desert neighborhood. Comparison: Neighborhood without change in existing supermarket facilities (three miles from the intervention neighborhood). Sub-group analyses on those adopting the store as their main store for grocery shopping compared to those that did not adopt the store (they did not use the store at all). Those that used the store as their secondary source of shopping were also compared to non-adopters. Sites were matched for race/ethnicity, income, demographics and size (3 miles^2^).	BMI (self-reported height and weight) Fruit and vegetable intake (self-report survey)	No significant changes were reported. *Time line changes*: There was a three-year delay in the construction of the supermarket.
**Studies without a control group**					
**Rail stops**					
Brown, B.B., and Werner, C.M. (2007) [33]. Salt Lake City, UT, USA Exposure: Rail stop	To test whether a new light-rail stop increases the number of light-rail riders and if light-rail ridership relates to moderate physical activity bouts.	Recruitment: Study notification letters delivered to addresses within ½ mile of the rail stop, followed by door-to-door recruitment. Incentive: $20 given for completing each phase. Participants: *N* = 529 (potential sample) living within half a mile of the new rail stop. Deemed ineligible = *n* = 33. Successfully contacted & invited, *n* = 215 (*n* = 102 agreed; *n* = 113 refused). T1 *n* = 102 (survey) and T2 *n* = 51 (survey) and *n* = 47 (accelerometer). Age (longitudinal sample): 41 ± 13.82 years. Time points: The rail-stop was added in autumn 2005. T1 = before summer 2005. T2 = after summer 2006 (1 year post T1).	Study design: pre-post WO control Exposure: building and opening of a new light-rail stop (between two existing stops) in the center of the surveyed neighborhood Comparison: No control group. Changes over time explored and associations between use of the light rail and physical activity.	Transit use—previous 2 weeks (self-report survey) MVPA bouts of ≥8 min over 7 days (accelerometer). MVPA discussed with participant to identify if it related to walking to/from the rail stop.	Rail use increased from 50% to 68.75% from T1 to T2 (*p* = 0.011). T1 MVPA was related to MVPA bouts at T2 (unstandardized beta coef = 0.38, SE = 0.12, *p* < 0.01). At T2 rail rides in the past 14 days (unstandardized beta coef = 0.03, SE = 0.01, *p* = 0.01) and bigger household sizes (unstandardized beta coef = 0.01, SE = 0.00, *p* = 0.01), account for variance beyond the effects of prior activity levels. No other significant changes were reported.
Hong, A., Boarnet, M.G. and Houston, D. (2016) [34].	To determine the impact of a new light rail transit line on active travel behavior	Recruitment: Invitation letters were sent to all households in the study area (*n* = 27,275). Incentive: $30 for T1 and $75 for T2 completion. Participants: The total sample at T1, was *n* = 279 (1% response rate, 74%F, aged 52 ± 14 years, 49% African-American) and at T2 was *n* = 204. Accelerometer and GPS data collected in *n* = 143 (66%F, aged 50 ± 14 years, 55% African-American) and analyzed for *n* = 73 participants. Time points: T1 = 5–7 months prior to the line opening. T2 = 2–6 months after the opening of the line.	Study design: pre-post WO control. Exposure: building a new light-rail line (with several stops). Comparison: No control group. Sub-group Changes over time explored in those residing <½ mile and >½ mile from the stations on the new line.	Transit usage and frequency of bus and train trips, frequency of walking and cycling (self-reported diary) Physical activity (accelerometer)	There was a negative association between total walk trips at T2 based on the interaction of distance to rail stop group and baseline walking trips (beta coef = −0.02, *p* = 0.008).
**Bike/walk trails**					
Burbidge, S.K., and Goulias, K.G. (2009) [35]. UT, USA Exposure: bike/walk trail	To determine the impact of introducing a neighborhood trail on active travel and total physical activity of residents.	Recruitment: NS. Participants: Activity diary component; *n* = 196 households (*n* = 175 individuals from *n* = 80 households at T1), *n* = 144 individuals from *n* = 56 households at T2 and *n* = 107 individuals from *n* = 41 household at T3). Questionnaire component; *n* = 290 households with 796 individuals living within 1 mile of the trail, plus a further 32 new resident households. Time points: The trail opened in September 2007. Activity diary; T1 = February 2007 (prior to trail construction), T2 = 1-month post trail opening (October 2007), T3 = 5-month post trail opening (February 2008). Questionnaire = October 2007.	Study design: longitudinal survey study WO control Exposure: building of a new trail along a canal route Comparison: No control group. Changes over time explored. Proximity to the trail on physical activity was also explored.	Single day activity data: activity type, begin and end time, activity duration, interpersonal interactions, travel related or not, distance travelled if any and mode used if travelled (Self-report activity diaries).	Data on residentially stable participants only reported here (data on new residents not reported). *t*-test: Total physical activity episodes (*p* = 0.036) and total walking trips (*p* = 0.008) decreased from T1 to T3. Regression: Total physical activity episodes (coef = −0.245, *p* = 0.036) and total walking trips (coef = −0.265, *p* = 0.008) decreased from T1 to T3. Regression after controlling for confounders: Total physical activity episodes increased from T1 to T3 in adults aged 18–64 years (*B* = 0.56, *p* = 0.024). No other significant changes were reported.
Evenson, K.R., et al., (2005) [36]. NC, USA Exposure: bike/walk trail	To explore changes in physical activity in local residents that might be attributable to the construction of a multi-use trail.	Recruitment: Approximately 28,304 people resided along the trail according to a census in 2000. A random list of 2125 households was generated from a telephone directory. Study postcards were mailed introducing the study followed by telephone surveys (<15 min) to residents living within two miles of the intervention site. The adult with the most recent birthday from each randomly selected household was invited to participate. Participants: *N* = 2125 adults from random households that had telephone numbers listed in the phone book were targeted from the 28,304 adults living in 11 census blocks that the trail traversed. *N =* 685 completed T1 surveys (47.2% response rate), *n* = 436 completed T2 surveys (63.7% retention; 4% refused a T2 survey). Final longitudinal sample: *n* = 366. Time points: The first segment of the trail (3.2 miles) opened in June 2000. The second segment (under investigation here) was 2.8 miles plus a 2.0 miles spur, opened in September 2002. T1 = July 2000–April 2001. T2 (1 year 7 months—2 years 4 months post-T1) = November 2002.	Study design: Pre-post WO control. Exposure: Building of a new walking and cycling trail. The new section of the trail passed by two schools, shopping areas, apartments, and neighborhood divisions and had several access points along the route. Comparison: No control group. Changes over time explored only in those that used the trail compared to those that did not use the trail were performed.	Leisure PA (self-report survey) Walking and cycling (self-report survey) MVPA (self-report survey) Transportation activity (self-report survey) Trail use (self-report survey) General health (self-report survey) BMI (self-reported height and weight)	Time in moderate PA (*p* = 0.03), time in vigorous PA (*p* < 0.0001) and cycling for transport (*p* = 0.01), decreased in those that reported not having used the trail. Time in vigorous PA (*p* = 0.01) decreased in those that had ever used the trail. Those that had used the trail were less likely to increase walking by >30 (OR = 0.46 (95% CI = 0.21–1.01)) or >45 min/week (OR = 0.43 (95% CI = 0.19–0.98)), and less likely to increase cycling by >30 (OR = 4.17 (95% CI = 1.70–10.20)), >15 (OR = 3.99 (95% CI = 1.81–8.79)) or >45 min (OR = 4.14 (95% CI = 1.33–12.90)) from baseline. No other changes were reported.
Goodman, A., et al. (2013) [37].^c^ Goodman, A., et al. (2014) [38].^d^ Cardiff, Kenilworth & Southampton, UK Exposure: bike/walk trail	To examine and compare patterns of use of high quality traffic free walking and cycling routes, including exploration of journey purpose for which routes were used and the modes by which it was used. Individual and household predictors of use are also determined. To determine the effects of new cycling and walking routes on overall physical activity levels, walking and cycling.	Recruitment: The electoral register was used to identify 22,500 adults living within 5 km of one of the sites. Surveys were mailed. Participants: Surveys were completed by *n* = 3516 adults at T1, *n* = 1885 adults at T2, and *n* = 1548 at T3. T2 comprised of *n* = 1849 (53% retention rate and 8% of the invited population) and T3 of *n* = 1510 (43% retention rate and 7% of the invited population) surveys. Physical activity data was collected in *n* = 1796 adults at T2 and *n* = 1465 adults at T3. Compared to local and national data, the sample had fewer young adults, were slightly healthier, better educated and less likely to have children than the general population. Time points: Most feeder routes were upgraded and the core projects had begun in Southampton and Cardiff in July 2010. By September 2011 the core Kenilworth project had begun and almost all feeder routes were complete. T1 = April 2010. T2 = 2011 (12-month follow up). T3 = 2012 (24-month follow-up). Baseline characteristics were measured in the 2010 questionnaire, and infrastructure use was measured in 2011.	Study design: pre-post WO control. Exposure: Building of new walking and cycling routes in three municipalities. Traffic-free bridges were built in Cardiff and Kenilworth, and a riverside footpath developed into a boardwalk in Southampton. Comparison: No control group. Changes over time explored. Changes based on distance to walk/cycle routes were included in the 2014 paper.	Use of new infrastructure, journey purpose and journey mode (self-report survey) Walking and cycling for different journey purposes (7-day recall) Recreational physical activity—total, moderate and vigorous intensity walking and cycling (IPAQ)	At T2 and T3, 32% and 38% of participants reported using the new infrastructure, respectively (change statistics over time NS and T1 values also NS). Walking for recreation was the most common use. Previous 7-day walking and cycling increased more from baseline in those living nearer to the exposures at T3 (adjusted effect = 15.3 min/week per km closer to the intervention; 95% CI = 6.5, 24.2; *p* < 0.001) in comparison to those living further from exposures. Proximity to exposure was strongly associated with total physical activity (12.5 min/week per km closer to the intervention; 95% CI = 1.9, 23.1). T3 effects of proximity were found for those reporting using routes (adjusted effect = 30.0 min/week; 95% CI 3.5, 55.5 in users) for total walking and cycling. Proximity to exposure was also associated with change in subdomains of physical activity at T3: cycling for recreation (adjusted effect = 2.5 min/week per km closer to the exposure; 95% CI = 0.1, 4.9); and walking for transport (adjusted effect = 8.8 min/week per km closer to the exposure; 95% CI = 2.8, 14.8). Change in walking and cycling was greater in those using the routes for ≥2 types of transport (adjusted effect = 46.4 min/week/km; 95% CI = 5.1, 87.7) compared to those using the route for <2 types of transport. Change in walking for recreation was greater in those reporting using the route for walking compared to those not reporting using the route for walking (adjusted effect = 33.3 min/week/km; 95% CI = 4.6, 62.0). Effects were attenuated but still significant in sensitivity analyses. No other significant changes were reported.
MacDonald, J.M., et al. (2010) [39]. Charlotte, NC Exposure: bike/walk trail	To determine the effect of using a light rail transit system on BMI, obesity and weekly physical activity.	Recruitment: Telephone sampling from census tract addresses within 1 mile of the new train line was undertaken. The adult with the most recent birthday was invited to participate. Overall response rate at T2 was 87% and 3% were refusals (*n* = 20). Participants: At T1 *n* = 839 (45% response rate) and at T2 *n* = 498 (60% of the T1 sample), adults participated. Only longitudinal sub-group analyses comparing users (*n* = 26) and non-users (*n* = 275) were reported; daily light-rail work commuters (*n* = 26 or 5.2%) compared with non-light rail users (*n* = 275). Time point: T1 (18 months prior to the opening of the system) = July 2006–February 2007. T2 = March–July 2008.	Study design: pre-post WO control. Exposure: Introduction of a new light rail transit system. Comparison: No control group. Changes over time explored in users versus non-users.	BMI and obesity (self-reported height and weight) Physical activity (self-report survey)	The exposure was associated with an average −1.18 (95% CI −2.22, −0.13) reduction in BMI (*p* < 0.05) and an 81% reduced odds (95% CI = 0.04, 0.92, *p* < 0.05) of becoming obese over time, in users compared to non-users. No other changes were reported.
Pazin, J., et al. (2016) [40]. Brazil Exposure: bike/walk trail	To examine the effects of a new cycling and walking route on physical activity in adults residing near the route.	Recruitment: Systematic sampling of households from lists of landlines, were used to identify individuals from six neighborhoods (*n* = 55,700) within 1500 m from the route (*n* = 7630). The first adult aged >18 years to answer a telephone invite was invited to participate. A sample size calculation was used and was fully reported on, to determine changes over time in the total sample (*n* = 656 participants were required). Participants: T1 = 745 (91% response rate from telephone invites and 10% of eligible individuals living in the neighborhoods). T2 = 519 (70% retention). Time points: T1 = March-July 2009. T2 = March–December 2012 (30 months post baseline).	Study design: Pre-post study WO control. Exposure: a new avenue, parking lots and a walking and cycling route, along a seashore. Comparison: No control group. Sub-groups consisting of residents that lived 0–500 m, 501–1000 m and 1001–1500 m from the route were compared.	Total weekly leisure time physical activity using questionnaire (IPAQ through telephone interview).	Leisure time walking increased, by 14 min/week (95% CI: 3–24) in residents. Leisure time walking increased by 32 min/week (95% CI: 15–51) in residents living up to 500 m from the new route, which was greater than in those living 501–1000 m away at follow-up (δ = 31 min/week; 95% CI: 11–51). Leisure time walking plus MVPA increased by 51 min/week (95% CI: 2–81) in those living up to 500 m from the new route. The percentage of participants that initiated leisure time walking or MVPA after the new route was negatively associated with the distance to the route. In participants that did not use the route, (*n* = 280), a greater proportion of residents in the –500 m (52%) and 501–1000 m (60%) groups reported intention to use the route compared to those in the 1001–1500 m (33%) group (*p* = 0.006). No other significant changes were reported.
Miller, H.J., et al. (2015) [41].^e^ Brown, B.B., et al. (2015) [42]. ^f^ US, Salt Lake City Exposure: bike/walk trail	To test if light rail transit (LRT) generated new PA in Salt Lake City, UT, USA. To assess effects on physical activity (PA) and weight among participants in a complete street intervention that extended a light-rail line in Salt Lake City, UT, USA.	Recruitment: Participants were recruited via door-to-door canvassing. Participants resided within 2 km of the new light rail transit line (exposure). Participants: *N* = 939 adults. A total of 614 participants completed 12-month follow-up, and 536 of these participants (51% female, 25% Hispanic) had valid Global Positioning System (GPS) data for analysis. Time points: T1: March–December 2012 and T2: May and November 2013 (the line opened in April 2013).	Study design: pre-post WO control. Exposure: building and opening of a new light-rail transit line. The transit line included the introduction of five additional residential stops a bike path and improved sidewalks in the area. Comparison: No control group. Changes over time explored. Sub-group analyses were completed: ‘Never’ (*N* = 391, including participants who had never used transit; used transit but not within the defined neighborhood; or only biked/walked in the neighborhood) ‘Continued’ (*N* = 51), ‘Former’ (*N* = 42, including those who had used transit during the first time period, but not the follow-up) ‘New’ (*N* = 52, including those with complete transit trips in follow-up, but not T1).	Physical activity (total and transit measured by GPS combined with accelerometer) BMI (researcher measured)	From T1 to T2, new riders increased transit physical activity by 3.46 min (95% CI: 2.20, 4.72, *p* < 0.0001). Former riders experienced a decrease of 2.34 min (95% CI: −3.56, −1.08, *p* = 0.0005) of transit physical activity. Accelerometer counts decreased in former riders from T1 to T2 (−49.35 ± 14.97 cpm; 95% CI: −78.75, −19.94), which was a greater change than in the never-riders, who slightly increased their accelerometer counts by 11.97 cpm, (*t* = −3.30; *p* = 0.001). New transit users accrued more accelerometer counts from T1 to T2 (37.40 ± 13.74 cpm; 95% CI: 10.41, 64.39) than never-riders (*t* = 2.72; *p* = 0.007). Former riders decreased MVPA minutes (−6.37 ± 2.01 min; 95% CI: −10.32, −2.43), which was different than the change in never riders from T1 to T2 (SE = 2.01; *t* = −3.17; *p* < 0.01). New riders increased MVPA by 4.16 ± 1.84 min; 95% CI: 0.54, 7.78), which was a bigger change than in the never riders (SE = 1.84; *t* = 2.26; *p* < 0.05). Sedentary behavior sig increased in the former riders by 16.38 ± 66.09 min; 95% CI: 4.41, 28.35, which was different than change in the never riders (SE = 6.09; *t* = 2.69; *p* < 0.01). In new riders, sedentary behavior decreased −12.83 ± 5.59 min; 95% CI: −23.82, −1.85, which was different to the change in the never riders (SE = 5.59; *t* = −2.30; *p* < 0.05). Former transit increased their BMI (0.64 ± 0.24 kg/m^2^ 95% CI: 0.18, 1.11), (*t* = 2.72; *p* = 0.007), whilst new riders had a decrease in BMI (−0.50 ± 0.22 kg/m^2^ 95% CI: −0.93, −0.08), (*t* = −2.32; *p* < 0.022). Both changes in former and new rider BMI were different than in never-riders, who had an increase in BMI of 0.19 kg/m^2^. Sensitivity analysis: All effects were sustained when 2012 baseline variables were included in analyses as a dependent variable as a predictor. One 1 new effect emerged: former riders had 11.34 fewer minutes of light PA than never-riders (*p* = 0.03).		
**Green space**							
West, S.T. & Shores, K.A. (2011) [43]. Southeastern U.S. Exposure: Green space	To determine if a new greenway increases physical activity levels in residents residing nearby.	Recruitment: The city planning department provided a list of property owners within one mile of the greenway (owning single-family units values >$5000). Invitation was random and via mail (study information letters and surveys were sent). Reminders were mailed 1-week later and another full package sent after the reminder. A total of 1168 invites were sent out. At T1 368/1168 replied (31.5% response rate). At T2 166/368 replied (45.1% response rate from T1 sample and 14.2% response rate from total invites sent out). Participants: Residents living within 0.5 miles = *n* = 597. Residents living within 0.5–1.0 miles = *n* = 571. Time points: The greenway was completed in early 2008. T1 = 2007. T2 (11 months after the intervention was complete) = 2008.	Study design: pre-post WO control Exposure: development of five miles of greenway (open space for recreation) alongside a river, which connects urban centers. Comparison: No control group. Changes over time explored. Sub-analyses on looking at differences between residents living within 0.5 miles compared to those living 0.51–1.0 miles from the greenway.	Physical activity, (self-report survey)	For the full sample, increases in days of walking for ≥30 min in the past week (2.9–3.3 days), participation in moderate PA (1.7–2.3 days) and participation in vigorous PA (1.3–1.8 days) increased (NS if changes were significant or not). Comparing those living <0.51 miles to those living 0.51–1.0 miles from the greenway, days of walking for ≥30 min in the past week (Eta^2^ = 0.53, *p* = 0.003), moderate activity (Eta^2^ = 0.133, *p* < 0.001) and vigorous activity (Eta^2^ = 1.47, *p* < 0.001) increased from T1 to T2. No interactions between greenway development and residential proximity were found for any measures. No other significant changes were reported.		
**Food retail**							
Evans, A.E., et al. (2012) [44]. Austin, TX, USA Exposure: farmers’ markets	To determine if introducing small farm stands without any other strategies in low-income communities increases fruit and vegetable intake in local residents.	Recruitment: Data collectors made door-to-door survey visits to low-income households within 0.5 miles of the stands at different times of day (recruitment goal was *n* = 100 adults). Streets were randomly selected for recruitment (all on the same side of the highway as the farm stands) and only houses perceived relatively safe were targeted (e.g., WO unleashed dogs). Only one attempt was made at each house. A total of *N* = 312 households were approached; *n* = 133 answered the door (43%) of total approached homes; *n* = 36 were not eligible or did not wish to participate. T2 data collection was over the telephone or via mail (if participant was not reached after five telephone call attempts). Six mail packets (8%) were undeliverable at T2 and 24 packets (51%) were not returned.	Study design: Pre-post WO control. Exposure: Two new farm stands introduced to a community (outside community centers 1 day/week for 12 weeks for 2–3 h each). Vouchers to assist low-income families to purchase healthy food were accepted by the stands. No advertisement of the stands occurred. No foods other than fruits and vegetables were available. Comparison: No control group. Changes over time explored.	Fruit and vegetable intake (self-report survey) Use of farm markets/stands (self-report survey)	Consumption of fruit (*p* < 0.001), fruit juice (*p* < 0.001), green salad (*p* < 0.05), tomatoes (*p* < 0.01) and other vegetables (*p* = 0.001) increased. Awareness of the market increased (from 19.3% to 39.3%, *p* = 0.001), as did purchasing of fruit and vegetables at the market (from 4.8% to 23.0%, *p* = 0.004). No other changes were reported.		
		Incentive: $10 gift cards were given to participants at T1 and T2. Participants: A total of *n* = 97 participated (*n* = 5 had missing data). Final T1 sample = *n* = 92. At T2 *n* = 47 or 51% of T1 participants completed the survey via telephone and *n* = 17 (36%) completed the survey via mail. Final longitudinal sample = *n* = 64. Time points: Intervention period was June-August 2010. T1 = May 2010. T2 = (2 months post-farm stand introduction) = July/August 2010.					
Wrigley, N., et al. (2002) [45].^g^ Wrigley, N., et al. (2003) [46].^h^ Leeds, UK Exposure: supermarket	To examine changes in food consumption and poverty after a sudden and significant change in food retail access. To explore the impact of a significant change in food retail provision in a highly deprived area on food consumption patterns	Recruitment: from a local authority housing estate area (population 38,000 and ~15,000 households). From a low income, socially deprived, largely white ethnicity background area. The main household domestic food purchaser was recruited. Target sample size was *n* = 1000 at T1 and *n* = 600 residents at T2. A target of inviting 3000 households at T1 was set. Incentives: vouchers for non-food related outlets were provided to participants at T1 and T2. Participants: T1 respondents = *n* = 1009. T2 respondents = *n* = 615. Primarily female participants (81.9% at T1 and 84.1% at T2). The non-respondents at T2 had moved residence (9%), could not be contacted after four attempts (13%) refused further participation (13%) and returned data that was unsatisfactory for inclusion (4%). Subgroup analyses in: participants eating ≤2 portion of fruit and vegetables/day at T1 that did switch (*n* = 124) and did not switch (*n* = 115), participants eating >2 to <3 portions at T1 that did (*n* = 52) and did not switch (*n* = 82) and those eating ≥3 portions at T1 that did (*n* = 100) and did not switch (*n* = 142); participants that switched to using the new supermarket at T2 (*n* = 276) compared to those that did not (*n* = 339); participants that switched to using the supermarket from using limited-range/budget stores (*n* = 48), a specific major retailer store (*n* = 110), other major retailer stores (*n* = 99, of which *n* = 87 were the same chain as the new supermarket) and other stores (*n* = 19) at T1 were compared to each other.	Study design: pre-post WO control. Exposure: New food retail in neighborhood. Comparison: Changes over time explored. (Sub-group exploration of: participants with poorest diets at baseline compared to others; those that switched to using the new facility compared to those that did not; those that stopped versus continued smoking; and those residing closer or further away from the facility).	Food consumption (self-report, 7-day diary) Interviewer administered surveys	Distance travelled to the main food store in those that switched to using the supermarket decreased from 2.25 to 0.98 km (statistics NS) from T1 to T2. In those that had shifted to using the supermarket, walking to the store as a mode of transportation increased from 12.3% to 30.8% and walking from the store increased from 6.5% to 22.8% (reported as significant in text, *p* = NS) from T1 to T2. Those that switched to using the supermarket increased fruit and vegetable consumption by 0.23 portions per day from T1 to T2 (*p* = 0.034). Participants eating ≤2 portions of fruit and vegetables/day at T1 that switched to using the new supermarket, increased fruit and vegetable consumption from T1 to T2 (from 1.25 to 1.72 portions/day, *p* < 0.001), but so did those that did not switch that were eating the same amount of fruit and vegetables at T1 (from 1.37 to 1.78 portions/day, *p* < 0.001). Those eating ≥3 portions of fruit and vegetables at T1 that did not switch to the supermarket had a decrease in fruit and vegetable intake (4.78–4.20 portions/day, *p* = 0.005). An area (area name = LS14 1) effect was found with participants living in one postcode area on fruit and vegetable intake at T2 (NS if this negative), but appears so from the table). All 2SLS and parameter estimates and OLS estimates had the same signs, with greater significance in relationships between fruit and vegetable intake and switching to using the supermarket, proximity to the supermarket and switching to using the supermarket from a limited-range/budget store at T1.		
		Participants living ≤750 m (*n* = 176) were compared to those that lived >750–≤1000 m (*n* = 113) and those that lived >1000 m (*n* = 326) from the supermarket and those living ≤500 m (*n* = 65) to those living >500–≤1000 m (*n* = 224) were also compared; participants that stopped smoking (*n* = 20) compared to those that did not; and participants living in different area codes. Multivariate analyses were conducted in *n* = 598 participants as *n* = 17 participants had missing information. Time points: Supermarket opening = November 2000. T1 (5 months before opening of the supermarket) = June–July 2000. T2 (7–8 months post-opening) = June–July 2001. The survey was piloted in February 2000. A repeatability survey was conducted during T2 of the main survey to examine random and systematic error extent (*n* = 140 households).			The effect of pre-intervention fruit and vegetable intake was less significant using 2SLS. Model 1 OLS parameter estimates and (SEs) for change in fruit and vegetable intake were: T1 fruit and vegetable consumption = −0.281 (0.034), *p* = 0.01); distance to supermarket ≤500 m = 0.440 (0.227), *p* = 0.05; switched to using supermarket from limited range/budget store in T1 = 0.386 (0.188), *p* = 0.05; and household within LS14 1 = −0.426 (0.160), *p* = 0.01. Model 2 OLS parameter estimates and (SEs) for change in fruit and vegetable intake were: T1 fruit and vegetable consumption = −0.282 (0.034), *p* = 0.01); and household within LS14 1 = −0.429 (0.159), *p* = 0.01. 2002 results: In those that had ‘poor’ diets at T1, fruit and vegetable intake increased by 60% (from 1.31 to 1.75 portions/day, with fruit/fruit-juice intake increasing by two-thirds nearly) and in those that had the ‘worst’ diets at T1, fruit and vegetable intake increased from 0.59 to 1.41 portions/day, with fruit/fruit-juice intake increasing five-fold (statistics NS). In those that were eating <1 portion/day at baseline, fruit and vegetable intake increased from 4.13 to 9.83 portions/week and fruit and fruit juice consumption increased from 0.77 to 3.92 portions/week, between T1 and T2. In those that were eating ≤2 portion/day of fruit and vegetables at baseline, fruit and vegetable intake rose from 9.17 to 12.25 portions/week and fruit and fruit juice intake increased from 2.82 to 4.59 portions/week (changes are stated as significant in text but significance values NS). In those completing T1 and T2 surveys, 45% switched to using the new supermarket as their main food retail source and 35% used the supermarket as their main fruit and vegetable source. In participants eating ≤2 portion/day of fruit and vegetables at baseline, 42% switched to using the new supermarket for fruit and vegetable purchasing. In participants eating <1 portion/day of fruit and vegetables at baseline, 70% switched to using the new supermarket for fruit and vegetable purchasing (significance of changes NS). No other significant changes were reported.		

NS = not stated; IPAQ = International Physical Activity Questionnaire; MVPA = moderate to vigorous physical activity; WO = without; T1 = time point 1 (baseline); T2 = follow-up 1 (post-baseline); T3 = follow-up 2 (post-follow-up 2); SD = standard deviation; SE = standard error; ‘±’ = SD; ~ = approximately; cpm = accelerometer counts per minute; OR = odds ratio; CI = confidence interval. All outcomes collected are listed in the outcome measures column. Only significant changes are reported in the efficacy column, where significance is set at *p* < 0.05 (e.g., if an outcome measure is not included in this column then there was no significant interaction effect (for RCTs) or change over time (for pre-post studies) for that outcome. Superscripts ^a–h^ indicate papers relating to the same study. Aims of each individual paper are provided directly in line with the respective reference details, whilst other data extracted common to both papers are not aligned (e.g., recruitment strategies and sample characteristics are the same for these papers).

**Table 3:** Risk of bias ratings for included studies.

Lead Author, Year, Reference	Item 1	Item 2	Item 3	Item 4	Item 5	Item 6	Item 7	Item 8	Item 9	Total
Brown, 2007, [33]	0	1	0	0	0	1	0	0	0	2
Burbridge, 2009, [35]	0	1	0	1	0	0	0	0	0	2
Cummins, 2005 & 2008, [30,31]	1	1	0	0	1	0	0	0	1	4
Cummins, 2014, [32]	1	0	1	0	1	0	1	0	1	5
Evans, 2012, [44]	0	1	0	1	0	0	0	0	0	2
Evenson, 2005, [36]	0	1	0	1	1	0	0	0	1	4
Goodman, 2013 and 2014, [37,38]	0	1	1	0	1	0	1	1	0	5
Macdonald, 2010, [39]	0	0	1	0	1	0	0	0	1	3
Quigg, 2011, [28]	1	0	0	1	1	1	1	0	0	5
West, 2011, [43]	0	0	0	0	0	0	0	1	0	1
Wrigley, 2002 & 2003, [45,46]	0	1	0	0	1	0	1	1	1	5
Dill, 2014, [29]	1	0	1	1	1	1	0	0	0	5
Miller, 2015, [41]	0	0	1	1	1	1	1	0	1	6
Pazin, 2016, [40]	0	1	1	1	1	0	1	1	0	6
Hong, 2016, [34]	0	0	0	1	1	1	0	1	0	4

1 = adequately addressed and reported; 0 = not addressed/not reported; Total = total number of items that were rated low risk (e.g., a higher number = lower risk of bias).

## Supplementary Materials

The following are available online at http://www.mdpi.com/1660-4601/15/2/217/s1, Table S1: Web of Knowledge search strategy and reasons for exclusion of articles, Table S2: Reasons for exclusion of articles at the final screening phase of the initial search in 2014.

## References

[1] Jackson R.J., Dannenberg A.L., Frumkin H. (2013). Health and the Built Environment: 10 Years After. Am. J. Public Health.

[2] Astell-Burt T., Feng X., Kolt G.S. (2014). Green space is associated with walking and moderate-to-vigorous physical activity (MVPA) in middle-to-older-aged adults: Findings from 203,883 Australians in the 45 and Up Study. Br. J. Sports Med..

[3] Duran A.C., de Almeida S.L., Latorre Mdo R., Jaime P.C. (2016). The role of the local retail food environment in fruit, vegetable and sugar-sweetened beverage consumption in Brazil. Public Health Nutr..

[4] Saelens B.E., Vernez Moudon A., Kang B., Hurvitz P.M., Zhou C. (2014). Relation Between Higher Physical Activity and Public Transit Use. Am. J. Public Health.

[5] Mertens L., Compernolle S., Deforche B., Mackenbach J.D., Lakerveld J., Brug J., Roda C., Feuillet T., Oppert J.M., Glonti K. (2017). Built environmental correlates of cycling for transport across Europe. Health Place.

[6] Ferdinand A.O., Sen B., Rahurkar S., Engler S., Menachemi N. (2012). The relationship between built environments and physical activity: A systematic review. Am. J. Public Health.

[7] Mackenbach J.D., Rutter H., Compernolle S., Glonti K., Oppert J.M., Charreire H., De Bourdeaudhuij I., Brug J., Nijpels G., Lakerveld J. (2014). Obesogenic environments: A systematic review of the association between the physical environment and adult weight status, the SPOTLIGHT project. BMC Public Health.

[8] Gong Y., Palmer S., Gallacher J., Marsden T., Fone D. (2016). A systematic review of the relationship between objective measurements of the urban environment and psychological distress. Environ. Int..

[9] Arcaya M.C., Tucker-Seeley R.D., Kim R., Schnake-Mahl A., So M., Subramanian S.V. (2016). Research on neighborhood effects on health in the United States: A systematic review of study characteristics. Soc. Sci. Med..

[10] Macintyre S. (2011). Good intentions and received wisdom are not good enough: The need for controlled trials in public health. J. Epidemiol. Community Health.

[11] Conway D., Li C.Q., Wolch J., Kahle C., Jerrett M. (2010). A Spatial Autocorrelation Approach for Examining the Effects of Urban Greenspace on Residential Property Values. J. Real Estate Financ. Econ..

[12] Bauman A.E., Reis R.S., Sallis J.F., Wells J.C., Loos R.J.F., Martin B.W., Lancet Physical Activity Series Working Group (2012). Correlates of physical activity: Why are some people physically active and others not?. Lancet.

[13] Petticrew M., Cummins S., Ferrell C., Findlay A., Higgins C., Hoy C., Kearns A., Sparks L. (2005). Natural experiments: An underused tool for public health?. Public Health.

[14] Pell J.P., Haw S., Cobbe S., Newby D.E., Pell A.C., Fischbacher C., McConnachie A., Pringle S., Murdoch D., Dunn F. (2008). Smoke-free Legislation and Hospitalizations for Acute Coronary Syndrome. NEJM.

[15] Astell-Burt T., Feng X., Kolt G.S., Jalaludin B. (2015). Does rising crime lead to increasing distress? Longitudinal analysis of a natural experiment with dynamic objective neighbourhood measures. Soc. Sci. Med..

[16] Goodman A., Panter J., Sharp S.J., Ogilvie D. (2013). Effectiveness and equity impacts of town-wide cycling initiatives in England: A longitudinal, controlled natural experimental study. Soc. Sci. Med..

[17] Galster G., Hedman L. (2013). Measuring Neighbourhood Effects Non-experimentally: How Much Do Alternative Methods Matter?. Hous. Stud..

[18] Feng J., Glass T.A., Curriero F.C., Stewart W.F., Schwartz B.S. (2010). The built environment and obesity: A systematic review of the epidemiologic evidence. Health Place.

[19] Kent J., Thompson S.M., Jalaludin B. (2011). Healthy Built Environments: A Review of the Literature.

[20] Smith M., Hosking J., Woodward A., Witten K., MacMillan A., Field A., Baas P., Mackie H. (2017). Systematic literature review of built environment effects on physical activity and active transport—An update and new findings on health equity. Int. J. Behav. Nutr. Phys. Act..

[21] Moher D., Liberati A., Tetzlaff J., Altman D.G., PRISMA Group (2009). Preferred reporting items for systematic reviews and meta-analyses: The PRISMA statement. J. Clin. Epidemiol..

[22] Petticrew M., Roberts H. (2006). Systematic Reviews in the Social Sciences: A Practical Guide.

[23] Benton J.S., Anderson J., Hunter R.F., French D.P. (2016). The effect of changing the built environment on physical activity: A quantitative review of the risk of bias in natural experiments. Int. J. Behav. Nutr. Phys. Act..

[24] Humphreys D.K., Panter J., Sahlqvist S., Goodman A., Ogilvie D. (2016). Changing the environment to improve population health: A framework for considering exposure in natural experimental studies. J. Epidemiol. Community Health.

[25] Higgins J.P.T., Green S. (2011). Cochrane Handbook for Systematic Reviews of Interventions Version 5.1.0.

[26] Craig P., Cooper C., Gunnell D., Haw S., Lawson K., Macintyre S., Ogilvie D., Petticrew M., Reeves B., Sutton M. (2012). Using natural experiments to evaluate population health interventions: New Medical Research Council guidance. J. Epidemiol. Community Health.

[27] Craig P., Cooper C., Gunnell D., Haw S., Lawson K., Macintyre S., Ogilvie D., Petticrew M., Reeves B., Sutton M. (2010). Using Natural Experiments to Evaluate Population Health Interventions: Guidance for Producers and Users of Evidence.

[28] Quigg R., Reeder A.I., Gray A., Holt A., Waters D. (2012). The Effectiveness of a Community Playground Intervention. J. Urban Health-Bull. N. Y. Acad. Med..

[29] Dill J., McNeil N., Broach J., Ma L. (2014). Bicycle boulevards and changes in physical activity and active transportation: Findings from a natural experiment. Prev. Med..

[30] Cummins S., Findlay A., Petticrew C.H.M., Sparks L., Thomson H. (2008). Reducing inequalities in health and diet: Findings from a study on the impact of a food retail development. Environ. Plan. A.

[31] Cummins S., Petticrew M., Higgins C., Findlay A., Sparks L. (2005). Large scale food retailing as an intervention for diet and health: Quasi-experimental evaluation of a natural experiment. J. Epidemiol. Community Health.

[32] Cummins S., Flint E., Matthews S.A. (2014). New neighborhood grocery store increased awareness of food access but did not alter dietary habits or obesity. Health Aff..

[33] Brown B.B., Werner C.M. (2007). A new rail stop: Tracking moderate physical activity bouts and ridership. Am. J. Prev. Med..

[34] Hong A., Boarnet M.G., Houston D. (2016). New light rail transit and active travel: A longitudinal study. Transp. Res. Part A Policy Pract..

[35] Burbidge S.K., Goulias K.G. (2009). Evaluating the Impact of Neighborhood Trail Development on Active Travel Behavior and Overall Physical Activity of Suburban Residents. Trans. Res. Rec..

[36] Evenson K.R., Herring A.H., Huston S.L. (2005). Evaluating change in physical activity with the building of a multi-use trail. Am. J. Prev. Med..

[37] Goodman A., Sahlqvist S., Ogilvie D. (2013). Who uses new walking and cycling infrastructure and how? Longitudinal results from the UK iConnect study. Prev. Med..

[38] Goodman A., Sahlqvist S., Ogilvie D., iConnect Consortium (2014). New walking and cycling routes and increased physical activity: One- and 2-year findings from the UK iConnect Study. Am. J. Public Health.

[39] MacDonald J.M., Stokes R.J., Cohen D.A., Kofner A., Ridgeway G.K. (2010). The effect of light rail transit on body mass index and physical activity. Am. J. Prev. Med..

[40] Pazin J., Garcia L.M., Florindo A.A., Peres M.A., Guimarães A.C., Borgatto A.F., Duarte Mde F. (2016). Effects of a new walking and cycling route on leisure-time physical activity of Brazilian adults: A longitudinal quasi-experiment. Health Place.

[41] Miller H.J., Tribby C.P., Brown B.B., Smith K.R., Werner C.M., Wolf J., Wilson L., Oliveira M.G. (2015). Public transit generates new physical activity: Evidence from individual GPS and accelerometer data before and after light rail construction in a neighborhood of Salt Lake City, Utah, USA. Health Place.

[42] Brown B.B., Werner C.M., Tribby C.P., Miller H.J., Smith K.R. (2015). Transit Use, Physical Activity, and Body Mass Index Changes: Objective Measures Associated with Complete Street Light-Rail Construction. Am. J. Public Health.

[43] West S.T., Shores K.A. (2011). The impacts of building a greenway on proximate residents' physical activity. J. Phys. Act. Health.

[44] Evans A.E., Jennings R., Smiley A.W., Medina J.L., Sharma S.V., Rutledge R., Stigler M.H., Hoelscher D.M. (2012). Introduction of farm stands in low-income communities increases fruit and vegetable among community residents. Health Place.

[45] Wrigley N., Warm D., Margetts B., Whelan A. (2002). Assessing the impact of improved retail access on diet in a food desert: A preliminary report. Urban Stud..

[46] Wrigley N., Warm D., Margetts B. (2003). Deprivation, diet, and food-retail access: Findings from the Leeds food deserts study. Environ. Plan. A.

[47] Australian Institute of Health and Welfare (2016). Health Behaviours and Their Role in the Prevention of Chronic Disease.

[48] Bonevski B., Randell M., Paul C., Chapman K., Twyman L., Bryant J., Brozek I., Hughes C. (2014). Reaching the hard-to-reach: A systematic review of strategies for improving health and medical research with socially disadvantaged groups. BMC Med. Res. Methodol..

[49] Booker C.L., Harding S., Benzeval M. (2011). A systematic review of the effect of retention methods in population-based cohort studies. BMC Public Health.

[50] Brueton V.C., Tierney J.F., Stenning S., Meredith S., Harding S., Nazareth I., Rait G. (2014). Strategies to improve retention in randomised trials: A Cochrane systematic review and meta-analysis. BMJ Open.

[51] Hunter R.F., Christian H., Veitch J., Astell-Burt T., Hipp J.A., Schipperijn J. (2015). The impact of interventions to promote physical activity in urban green space: A systematic review and recommendations for future research. Soc. Sci. Med..

[52] Green J., Steinbach R., Jones A., Edwards P., Kelly C., Nellthorp J., Goodman A., Roberts H., Petticrew M., Wilkinson P. (2014). On the Buses: A Mixed-Method Evaluation of the Impact of Free Bus Travel for Young People on the Public Health.

[53] Brownson R.C., Baker E.A., Boyd R.L., Caito N.M., Duggan K., Housemann R.A., Kreuter M.W., Mitchell T., Motton F., Pulley C. (2004). A community-based approach to promoting walking in rural areas. Am. J. Prev. Med..

[54] Merom D., Bauman A., Vita P., Close G. (2003). An environmental intervention to promote walking and cycling—The impact of a newly constructed Rail Trail in Western Sydney. Prev. Med..

[55] Wiehe S.E., Carroll A.E., Liu G.C., Haberkorn K.L., Hoch S.C., Wilson J.S., Fortenberry J.D. (2008). Using GPS-enabled cell phones to track the travel patterns of adolescents. Int. J. Health Geogr..

[56] Farrukh M., Farooq T.A., Saeed A., Sultan F., Inam A., Afzal M., Nadeem U., Khan S.A. (2014). Working Paper: Using Smart Phones to Monitor Attendance in Public Facilities.

[57] Ogilvie D., Bull F., Cooper A., Rutter H., Adams E., Brand C., Ghali K., Jones T., Mutrie N., Powell J. (2012). Evaluating the travel, physical activity and carbon impacts of a ‘natural experiment’ in the provision of new walking and cycling infrastructure: Methods for the core module of the iConnect study. BMJ Open.

[58] Zhang Y.T., Laraia B.A., Mujahid M.S., Blanchard S.D., Warton E.M., Moffet H.H., Karter A.J. (2016). Is a reduction in distance to nearest supermarket associated with BMI change among type 2 diabetes patients?. Health Place.

[59] Schröer S., Haupt J., Pieper C. (2014). Evidence-based lifestyle interventions in the workplace: An overview. Occup. Med..

[60] Kirk S.F.L., Penney T.L., McHugh T.L., Sharma A.M. (2012). Effective weight management practice: A review of the lifestyle intervention evidence. Int. J. Obes..

[61] Rose G. (2001). Sick individuals and sick populations. Int. J. Epidemiol..

