# Supplementary material for: Do Natural Experiments of Changes in Neighborhood Built Environment Impact Physical Activity and Diet? A Systematic Review
Source: Int J Environ Res Public Health. 2018 Jan 26;15(2):217. doi: 10.3390/ijerph15020217 (PMC5858286; doi:10.3390/ijerph15020217)
Supplement: Supplementary file 1 [file ijerph-15-00217-s001.pdf]

Table S1: Web of Knowledge search strategy and reasons for exclusion of articles

#17	#16 AND #12 AND #5<br>*DocType=All document types; Language=All languages;*
#16	TS=("built environment*" OR "environment* design" OR "residential environment*" OR "neighbourhood environment" OR “neighborhood environment" OR “residen* characteristic$" OR "residen* facilit*" OR “local environment$" OR migration OR “food retail access” OR "neighborhood effect$" OR “retail-provision intervention” OR “retail intervention” OR “environment* change” OR “multi-use trail” OR “environmental-factors” OR “park improvements” OR park$ OR “grocery store$” OR “food access*” OR “food retail*” OR “residential segregation” OR “housing vouchers” OR “outdoor exercise equipment” OR “urban public park$” OR “home environment” or “local neighbourhood” OR “local neighborhood” OR “urban sprawl” OR segregation)<br>*DocType=All document types; Language=All languages;*
#15	#14 AND #12 AND #1<br>*DocType=All document types; Language=All languages;*
#14	TS=(“health behavior" OR “health behaviour" OR "health behaviors" OR "health behaviours" OR "quality of life" OR lifestyle OR "physical activit*" OR diet OR “body mass index” OR inactivity OR “physical fitness” OR “health promotion” or obesity or “well-being” OR “park use” OR "physical health" OR "mental health")<br>*DocType=All document types; Language=All languages;*
#13	#12 AND #5 AND #1<br>*DocType=All document types; Language=All languages;*
#12	TS=("experimental design" OR "before/after study" OR "panel stud*" OR "natural experiment" OR "natural experiment* stud*" OR "quasi experiment* stud*" OR "longitudinal stud*" OR “pre- and post-intervention” OR “pre-post design” OR “prospective evaluation$” OR “intervention community” OR “community level intervention$” OR "social experiment" OR longitudinal OR "housing mobility experiment")<br>*DocType=All document types; Language=All languages;*
#11	#10 AND #5 AND #1<br>*DocType=All document types; Language=All languages;*
#10	TS=("experimental design" OR "before/after study" OR "panel stud*" OR "natural experiment" OR "natural experiment* stud*" OR "quasi experiment* stud*" OR "longitudinal stud*" OR “pre- and post-intervention” OR “pre-post design” OR “prospective evaluation$” OR “intervention community” OR “community level intervention$” OR "social experiment" OR longitudinal)<br>*DocType=All document types; Language=All languages;*
#9	#6 AND #4 AND #3<br>*DocType=All document types; Language=All languages;*
#8	#6 AND #5 AND #2<br>*DocType=All document types; Language=All languages;*
#7	#5 AND #4 AND #1<br>*DocType=All document types; Language=All languages;*
#6	TS=("built environment*" OR "environment* design" OR "residential environment*" OR "neighbourhood environment" OR “neighborhood environment" OR “residen* characteristic$" OR "residen* facilit*" OR “local environment$" OR migration OR “food retail access” OR "neighborhood effect$" OR “retail-provision intervention” OR “retail intervention” OR “environment* change” OR “multi-use trail” OR “environmental-factors” OR “park improvements” OR park$)<br>*DocType=All document types; Language=All languages;*
#5	TS=(“health behavior" OR “health behaviour" OR "health behaviors" OR "health behaviours" OR "quality of life" OR lifestyle OR "physical activit*" OR diet OR “body mass index” OR inactivity OR “physical fitness” OR “health promotion” or obesity or “well-being” OR “park use”)<br>*DocType=All document types; Language=All languages;*
#4	TS=("experimental design" OR "before/after study" OR "panel stud*" OR "natural experiment" OR "natural experiment* stud*" OR "quasi experiment* stud*" OR "longitudinal stud*" OR “pre- and post-intervention” OR “pre-post design” OR “prospective evaluation$” OR “intervention community” OR “community level intervention$”)

Table S2: Reasons for exclusion of articles at the final screening phase of articles from database searching (n=82 from the initial search in 2014 and n=18 from the updated search in 2017 indicated by *)

Code classification for exclusions

- Not a pre-post, experimental study with a specific change in built environment or paper does not report findings – 1
- Not local residents or residentially stable participants – 2
- No beneficial change/s in public built environment in a residential setting (e.g. not work place settings) – 3
- Multi-component intervention – 4
- Not in English or unavailable in Australia – 5
- No physical activity or diet outcome measure - 6

Reference	Reason for exclusion	Code
*Airaksinen, J., Hakulinen, C., Elovainio, M., Lehtimaki, T., Raitakari, O. T., Keltikangas-Jarvinen, L., & Jokela, M. (2015). Neighborhood effects in depressive symptoms, social support, and mistrust: Longitudinal analysis with repeated measurements. *Social Science & Medicine, 136*, 10-16. doi:10.1016/j.socscimed.2015.04.034	Exclude – no measure of physical activity or diet	6
Bacigalupe, A., Esnaola, S., Calderon, C., Zuazagoitia, J., & Aldasoro, E. (2010). Health impact assessment of an urban regeneration project: opportunities and challenges in the context of a southern European city. *J Epidemiol Community Health, 64*(11), 950-955	Exclude – only one set of interviews conducted at baseline, not longitudinal	1
Barnes, G., Thompson, K., & Krizek, K. (2006). A longitudinal analysis of the effect of bicycle facilities on commute mode share. *Transportation Research Board 85th Annual Meeting.*	Exclude – census data used, not the same participants at baseline and follow-up. Conference paper.	2
Blackman, T., & Harvey, J. (2001). Housing renewal and mental health: a case study. *Journal of Mental Health, 10*(5), 571-583.	Exclude – multi-component (includes household infrastructure regeneration as well as neighbourhood)	4
Bond, L., Egan, M., Kearns, A., Clark, J., & Tannahill, C. (2013). Smoking and intention to quit in deprived areas of Glasgow: is it related to housing improvements and neighbourhood regeneration because of improved mental health? *Journal of Epidemiology & Community Health, 67*(4), 299-304.	Exclude – changes are at the individual housing level and not the public infrastructure level	3

Branas, C.C., Cheney, R.A., MacDonald, J.M., Tam, V.W., Jackson, T.D., Ten Have, T.R. (2011). A difference-in-differences analysis of health, safety, and greening vacant urban space. *American Journal of Epidemiology, 174* (11), 1296-1306.	Exclude – not the same participants at each time point	2
*Brand, C., Goodman, A., Ogilvie D., & iConnect, C. (2014). Evaluating the impacts of new walking and cycling infrastructure on carbon dioxide emissions from motorized travel: A controlled longitudinal study. *Applied Energy, 128*, 284-295. doi:10.1016/j.apenergy.2014.04.072	Exclude - primary outcome carbon dioxide emissions. The main outcome paper focusing on physical activity is included in the review	6
Bohn-Goldbaum, E. E., Phongsavan, P., Merom, D., Rogers, K., Kamalesh, V., & Bauman, A. E. (2013). Does playground improvement increase physical activity among children? A quasi-experimental study of a natural experiment. *J Environ Public Health, 2013*, 109841.	Exclude - participants do not specifically include local residents (only park users)	2
Brown, A. L., Khattak, A. J., & Rodriguez, D. A. (2008). Neighbourhood types, travel and body mass: A study of new urbanist and suburban neighbourhoods in the US. *Urban Studies, 45*(4), 963-988.	Exclude – cross-sectional	1
Casciano, R., & Massey, D. S. (2012). Neighborhood disorder and anxiety symptoms: new evidence from a quasi-experimental study. *Health & Place, 18*(2), 180-190.	Exclude - Comparing groups but no changes in built environment	3
Cerda, M., Morenoff, J. D., Hansen, B. B., Tessari Hicks, K. J., Duque, L. F., Restrepo, A., et al. (2012). Reducing violence by transforming neighborhoods: a natural experiment in Medellin, Colombia. *Am J Epidemiol, 175*(10), 1045-1053.	Exclude – outcome measure not health (violence)	6
Chang, C. H., Lu, M. S., Lin, T. E., & Chen, C. H. (2013). The Effectiveness of Visual Art on Environment in Nursing Home. *Journal of Nursing Scholarship, 45*(2), 107-115.	Exclude – change within nursing home rather than the neighbourhood	3
*Chen, H. J., & Wang, Y. (2014). Gender differences in impact of food stores in neighborhoods on children's obesity status change: U.S. Early childhood longitudinal study. *FASEB Journal. Conference: Experimental Biology, 28*(1 SUPPL. 1).	Exclude – no measure of physical activity or diet included	6
Ciaranello, A. L., Molitor, F., Leamon, M., Kuenneth, C., Tancredi, D., Diamant, A. L., & Kravitz, R. L. (2006). Providing health care services to the formerly homeless: a quasi-experimental evaluation. *J Health Care Poor Underserved, 17*(2), 441-461. doi: 10.1353/hpu.2006.0056.	Exclude – not a built environment intervention (human resources intervention)	3

Cohen, D. A., Marsh, T., Williamson, S., Golinelli, D., & McKenzie, T. L. (2012). Impact and cost-effectiveness of family Fitness Zones: A natural experiment in urban public parks. *Health & Place, 18*(1), 39-45.	Exclude - participants do not specifically include local residents (only park users)	2
Cohen, D. A., Sehgal, A., Williamson, S., Marsh, T., Golinelli, D., & McKenzie, T. L. (2009). New Recreational Facilities for the Young and the Old in Los Angeles: Policy and Programming Implications. *Journal of Public Health Policy, 30*, S248-S263.	Exclude – do not state if the same participants were included at T2 as T1	2
Cohen, D. A., Han, B., Derose, K. P., Williamson, S., Marsh, T., & McKenzie, T. L. (2013). Physical activity in parks: A randomized controlled trial using community engagement. *Am J Prev Med, 45*(5), 590-597.	Exclude – multi-component	4
Cohen, D. A., Golinelli, D., Williamson, S., Sehgal, A., Marsh, T., & McKenzie, T. L. (2009). Effects of Park Improvements on Park Use and Physical Activity Policy and Programming Implications. *American Journal of Preventive Medicine, 37*(6), 475-480	Exclude – multi-component as community involvement was included in the design and oversight of the development	4
Cohen, D. A., et al. (2014). The potential for pocket parks to increase physical activity. *American Journal of Health Promotion 28*(3): S19-S26.	Exclude – longitudinal findings not reported separate to cross-sectional data (cross-sectional added to the longitudinal data)	1
*Cranney, L., Phongsavan, P., Kariuki, M., Stride, V., Scott, A., Hua, M., & Bauman, A. (2015). Impact of an outdoor gym on park users' physical activity: A natural experiment. *Health Place, 37*, 26-34. doi:10.1016/j.healthplace.2015.11.002	Exclude – did not include residentially stable participants (random intercept data collection was used at each time point)	2
*Dill, J., McNeil, N., Broach, J., & Ma, L. (2014). Bicycle boulevards and changes in physical activity and active transportation: findings from a natural experiment. *Preventive Medicine, 69*, S74-S78. doi:http://dx.doi.org/10.1016/j.ypmed.2014.10.006	Exclude – not one specific change in built environment as the focus of the intervention (time varying changes in infrastructure included)	1
Donovan, G. H., Butry, D. T., Michael, Y. L., Prestemon, J. P., Liebhold, A. M., Gatziolis, D., & Mao, M. Y. (2013). The Relationship Between Trees and Human Health Evidence from the Spread of the Emerald Ash Borer. *American Journal of Preventive Medicine, 44*(2), 139-145.	Exclude – natural rather than built environment	3

*Dubowitz, T., Ghosh-Dastidar, M., Cohen, D. A., Beckman, R., Steiner, E. D., Hunter, G. P., . . . Collins, R. L. (2015). Diet And Perceptions Change With Supermarket Introduction In A Food Desert, But Not Because Of Supermarket Use. *Health Aff (Millwood), 34*(11), 1858-1868. doi:10.1377/hlthaff.2015.0667	Exclude – public discussions and marketing campaigns on healthy foods accompanied the built environment change	4
*Dubowitz, T., Ncube, C., Leuschner, K., & Tharp-Gilliam, S. (2015). A natural experiment opportunity in two low-income urban food desert communities: research design, community engagement methods, and baseline results. *Health Education & Behavior, 42*(1 Suppl), 87S-96S. doi:http://dx.doi.org/10.1177/1090198115570048	Exclude – no longitudinal data included	1
Egan, M., Katikireddi, S. V., Kearns, A., Tannahill, C., Kalacs, M., & Bond, L. (2013). Health effects of neighborhood demolition and housing improvement: a prospective controlled study of 2 natural experiments in urban renewal. *Am J Public Health, 103*(6), e47-53.	Exclude – focuses on the negative rather than positive effects of changes in infrastructure on health (impacts of demolition)	3
el-Askari, G., Freestone, J., Irizarry, C., Kraut, K. L., Mashiyama, S. T., Morgan, M. A., & Walton, S. (1998). The Healthy Neighborhoods Project: a local health department's role in catalyzing community development. *Health Educ Behav, 25*(2), 146-159.	Exclude – descriptive paper	1
*Elbel, B., Mijanovich, T., Kiszko, K., Abrams, C., Cantor, J., & Dixon, L. B. (2015). The Introduction of a Supermarket via Tax-Credits in a Low-Income Area: The Influence on Purchasing and Consumption. *Am J Health Promot*. doi:10.4278/ajhp.150217-QUAN-733	Exclude – local intercept data collection (not the same residents at baseline and follow-ups)	2
*Elbel, B., Moran, A., Dixon, L. B., Kiszko, K., Cantor, J., Abrams, C., & Mijanovich, T. (2015). Assessment of a government-subsidized supermarket in a high-need area on household food availability and children's dietary intakes. *Public Health Nutrition, 18*(15), 2881-2890. doi:10.1017/s1368980015000282	Exclude – local intercept data collection (not the same residents at baseline and follow-ups)	2
Fitzhugh, E. C., Bassett Jr, D. R., & Evans, M. F. (2010). Urban trails and physical activity: A natural experiment. *American Journal of Preventive Medicine, 39*(3), 259-262.	Exclude – local residents were not specifically recruited (direct observation of neighbourhood areas)	2

*Foley, L., Panter, J., Heinen, E., Prins, R., & Ogilvie, D. (2015). Changes in active commuting and changes in physical activity in adults: a cohort study. *Int J Behav Nutr Phys Act, 12*, 161. doi:10.1186/s12966-015-0323-0	Exclude – the intervention was in tandem with other interventions including town-level initiatives to encourage more and safer cycling	4
Foster, S., Knuiman, M., Hooper, P., Christian, H., & Giles-Corti, B. (2014). Do changes in residents' fear of crime impact their walking? Longitudinal results from RESIDE. *Prev Med, 62*, 161-166.	Exclude – data was collected 18 and 24 months after participants moved into their new homes but not including the same residents at both time points	2
Frankenberg, E., Nobles, J., & Sumantri, C. (2012). Community destruction and traumatic stress in post-tsunami Indonesia. *J Health Soc Behav, 53*(4), 498-514. doi: 10.1177/0022146512456207.	Exclude – natural disaster and not a built environment intervention	3
Goodman, A., Panter, J., Sharp, S. J., & Ogilvie, D. (2013). Effectiveness and equity impacts of town-wide cycling initiatives in England: A longitudinal, controlled natural experimental study. *Social Science & Medicine, 97*, 228-237.	Exclude- multi-component	4
Goldsby, T. U., et al. (2016). Urban park development and pediatric obesity rates: A quasi-experiment using electronic health record data. International Journal of Environmental Research and Public Health *13*(4): 411-421.	Exclude – no measure of physical activity or diet	6
Handy, S. L., Cao, X. Y., & Mokhtarian, P. L. (2007). The causal influence of neighbourhood design on physical activity within the neighbourhood: Evidence from Northern California. *American Journal of Health Promotion, 22*(5), 350-358.	Exclude – observational not experimental	1
*Heinen, E., Panter, J., Mackett, R., & Ogilvie, D. (2015). Changes in mode of travel to work: a natural experimental study of new transport infrastructure. *International Journal of Behavioral Nutrition and Physical Activity, 12*, 10. doi:10.1186/s12966-015-0239-8	Exclude – the intervention was in tandem with other interventions including town-level initiatives to encourage more and safer cycling	4
*Heinen, E., Panter, J., Dalton, A., Jones, A., & Ogilvie, D. (2015). Sociospatial patterning of the use of new transport infrastructure: Walking, cycling and bus travel on the Cambridgeshire guided busway. *Journal of Transport & Health, 2*(2), 199-211. doi:10.1016/j.jth.2014.10.006	Exclude – the intervention was in tandem with other interventions including town-level initiatives to encourage more and safer cycling	4

Hirsch, J. A., et al. (2014). Built environment change and change in BMI and waist circumference: Multi-ethnic study of atherosclerosis. *Obesity,* 22(11), 2450-2456.	Exclude – time-varying design	1
Hirsch, J. A., et al. (2014). Changes in the built environment and changes in the amount of walking over time: Longitudinal results from the Multi-Ethnic Study of Atherosclerosis. *American Journal of Epidmiology,* 180(8), 799-809.	Exclude – time-varying design	1
Hunter, R., Tully, M., & Kee, F. (2012). Development of an evaluation framework to measure the public health impact of a 'natural experiment: The PARC study. *Journal of Science and Medicine in Sport, 15*, S309.	Exclude – protocol paper	1
Jackson, M. I., & Mare, R. D. (2007). Cross-sectional and longitudinal measurements of neighborhood experience and their effects on children. *Social Science Research, 36*(2), 590-610.	Exclude – observational not experimental	1
Jilcott, S. B., Laraia, B. A., Evenson, K. R., Lowenstein, L. M., & Ammerman, A. S. (2007). A guide for developing intervention tools addressing environmental factors to improve diet and physical activity. *Health Promotion Practice, 8*(2), 192-204.	Exclude – paper on the development of a research tool	1
Johansson, P., Sadigh, S., Tillgren, P., & Rehnberg, C. (2008). Non-pharmaceutical prevention of hip fractures - A cost-effectiveness analysis of a community-based elderly safety promotion program in Sweden. *Cost Effectiveness and Resource Allocation, 6*(11).	Exclude – multi-component intervention	4
Johnson, F. R., & Luken, R. A. (1987). Radon risk information and voluntary protection: evidence from a natural experiment. *Risk Anal, 7*(1), 97-107.	Exclude – not a built environment related intervention	3
Johnston, Y. A., McFadden, M., Lamphere, M., Buch, K., Stark, B., & Salton, J. L. (2014). Working with grocers to reduce dietary sodium: lessons learned from the Broome County Sodium Reduction in Communities pilot project. *J Public Health Manag Pract, 20*(1 Suppl 1), S54-58. doi: 10.1097/PHH.0b013e3182a0b91a.	Exclude – descriptive paper	1
Kamanda, A., Embleton, L., Ayuku, D., Atwoli, L., Gisore, P., Ayaya, S., . . . Braitstein, P. (2013). Harnessing the power of the grassroots to conduct public health research in sub-Saharan Africa: a	Exclude – not a built environment intervention	3

case study from western Kenya in the adaptation of community-based participatory research (CBPR) approaches. *BMC Public Health, 13*(91).		
Kramer, D., Droomers, M., Jongeneel-Grimen, B., Wingen, M., Stronks, K., & Kunst, A. E. (2014). The impact of area-based initiatives on physical activity trends in deprived areas; a quasi-experimental evaluation of the Dutch District Approach. *International Journal of Behavioral Nutrition and Physical Activity, 11*.	Exclude – multi-component	4
Kegler, M. C., Alcantara, I., Veluswamy, J. K., Haardorfer, R., Hotz, J. A., & Glanz, K. (2012). Results From an Intervention to Improve Rural Home Food and Physical Activity Environments. *Progress in Community Health Partnerships-Research Education and Action, 6*(3), 265-277.	Exclude – not a built environment intervention	3
Keltner, B. R. (1992). Family influences on child health status. *Pediatric Nursing, 18*(2), 128-131.	Exclude – not a built environment intervention	3
Kindt, K. C., van Zundert, R., & Engels, R. C. (2012). Evaluation of a Dutch school-based depression prevention program for youths in high-risk neighborhoods: study protocol of a two-armed randomized controlled trial. *BMC Public Health, 12*, 212. doi: 10.1186/1471-2458-12-212.	Exclude – not a change in the neighborhood built environment (school-based)	3
King, D. K., Glasgow, R. E., & Leeman-Castillo, B. (2010). Reaiming RE-AIM: using the model to plan, implement, and evaluate the effects of environmental change approaches to enhancing population health. *American Journal of Public Health, 100*(11), 2076-2084. doi: 10.2105/AJPH.2009.190959.	Exclude – description of how to evaluate natural experiments	1
Kohlmeier, L. (1991). Food patterns and health problems: central Europe. *Ann Nutr Metab, 35 Suppl 1*, 22-31.	Exclude – not experimental	1
Lamarca, Gabriela A., Leal, Maria do C., Sheiham, Aubrey, & Vettore, Mario V. (2013). The association of neighbourhood and individual social capital with consistent self-rated health: a longitudinal study in Brazilian pregnant and postpartum women. *BMC Pregnancy & Childbirth, 13*, 1-1. doi: 10.1186/1471-2393-13-1.	Exclude – not experimental	1
Leatherdale, S. T., Brown, K. S., Carson, V., Childs, R. A., Dubin, J. A., Elliott, S. J., . . . Thompson-Haile, A. (2014). The COMPASS study: a longitudinal hierarchical research platform for evaluating	Exclude – multi-component	4

natural experiments related to changes in school-level programs, policies and built environment resources. *BMC Public Health, 14*(1), 331. doi: 10.1186/1471-2458-14-331.		
Lee, R. E., Mama, S. K., & Adamus-Leach, H. J. (2012). Neighborhood street scale elements, sedentary time and cardiometabolic risk factors in inactive ethnic minority women. *PLoS ONE [Electronic Resource], 7*(12).	Exclude – observational not experimental	1
Lee, R. E., Mama, S. K., Medina, A. V., Ho, A., & Adamus, H. J. (2012). Neighborhood factors influence physical activity among African American and Hispanic or Latina women. *Health & Place, 18*(1), 63-70.	Exclude – observational not experimental	1
Linenger, J. M., Chesson, C. V., & Nice, D. S. (1991). Physical-Fitness Gains Following Simple Environmental-Change. *American Journal of Preventive Medicine, 7*(5), 298-310.	Exclude – set in a naval base	3
Lovato, C., Watts, A., Brown, K. S., Lee, D., Sabiston, C., Nykiforuk, C., . . . Thompson, M. (2013). School and community predictors of smoking: a longitudinal study of Canadian high schools. *Am J Public Health, 103*(2), 362-368. doi: 10.2105/ajph.2012.300922.	Exclude – not experimental	1
Maibach, E. W. (2003). Recreating communities to support active living: a new role for social marketing. *American Journal of Health Promotion, 18*(1), 114-119.	Exclude – descriptive study	1
*Mehdipanah, R., Rodriguez-Sanz, M., Malmusi, D., Muntaner, C., Diez, E., Bartoll, X., & Borrell, C. (2014). The effects of an urban renewal project on health and health inequalities: a quasi-experimental study in Barcelona. *Journal of Epidemiology & Community Health, 68*(9), 811-817. doi:http://dx.doi.org/10.1136/jech-2013-203434	Exclude – repeated cross-sectional surveys (not the same participants at baseline and follow-ups)	2
Michael, Y. L., et al. (2014). Does change in the neighbourhood environment prevent obesity in older women? *Social Science & Medicine, 102*: 129-137.	Exclude – time-varying design	1
Millar, L., Kremer, P., De Silva-Sanigorski, A., Mathews, L., Malakellis, M., Robertson, N., . . . Swinburn, B. (2011). Behavioural and environmental changes and concomitant increases in schools' capacity during the it's your move! Adolescent, community-based obesity prevention intervention. *Obesity Reviews, 12*, 67.	Exclude – not a built environment intervention	3

Mohnen, Sigrid M., Völker, Beate, Flap, Henk, Subramanian, S. V., & Groenewegen, Peter P. (2013). You have to be there to enjoy it? Neighbourhood social capital and health. *European Journal of Public Health, 23*(1), 33-39. doi: 10.1093/eurpub/cks039.	Exclude – not a built environment intervention	3
Norback, D., Zock, J. P., Plana, E., Heinrich, J., Svanes, C., Sunyer, J., . . . Jarvis, D. (2011). Lung function decline in relation to mould and dampness in the home: the longitudinal European Community Respiratory Health Survey ECRHS II. *Thorax, 66*(5), 396-401. doi: 10.1136/thx.2010.146613.	Exclude – observational not experimental	1
Ogilvie, D., Griffin, S., Jones, A., Mackett, R., Guell, C., Panter, J., et al. (2010). Commuting and health in Cambridge: a study of a 'natural experiment' in the provision of new transport infrastructure. *BMC public health, 10*, 703.	Exclude – protocol paper	1
*Panter, J., Griffin, S., & Ogilvie, D. (2014). Active commuting and perceptions of the route environment: a longitudinal analysis. *Preventive Medicine, 67*, 134-140. doi:http://dx.doi.org/10.1016/j.ypmed.2014.06.033	Exclude – the intervention was in tandem with other interventions including town-level initiatives to encourage more and safer cycling	4
*Panter, J., Heinen, E., Mackett, R., & Ogilvie, D. (2016). Impact of New Transport Infrastructure on Walking, Cycling, and Physical Activity. *American Journal of Preventive Medicine, 50*(2), e45-e53. doi:https://doi.org/10.1016/j.amepre.2015.09.021	Exclude – the intervention was in tandem with other interventions including town-level initiatives to encourage more and safer cycling	4
Petersen, K. L., Nicholls, T. L., Groden, D., Schmitz, N., Stip, E., Goldner, E. M., . . . Lesage, A. (2013). Redevelopment of tertiary psychiatric services in British Columbia: A prospective study of clinical, social, and residential outcomes of former long-stay inpatients. *Schizophrenia Research, 149*(1-3), 96-103.	Exclude – descriptive paper	1
Pettersson, C., Lofqvist, C., & Fange, A. M. (1706). Clients' experiences of housing adaptations: a longitudinal mixed-methods study. *Disability & Rehabilitation, 34*(20), 1706-1715.	Exclude – change within homes of participants	3
Pribesh, S., & Downey, D. B. (1999). Why are residential and school moves associated with poor school performance? *Demography, 36*(4), 521-534.	Exclude – theoretical paper	1

*Ranchod, Y. K., Diez Roux, A. V., Evenson, K. R., Sanchez, B. N., & Moore, K. (2014). Longitudinal associations between neighborhood recreational facilities and change in recreational physical activity in the multi-ethnic study of atherosclerosis, 2000-2007. *American Journal of Epidemiology, 179*(3), 335-343. doi:http://dx.doi.org/10.1093/aje/kwt263	Exclude – time-varying design	1
Reed, Julian, Price, Anna, Grost, Lisa, & Mantinan, Karah. (2012). Demographic Characteristics and Physical Activity Behaviors in Sixteen Michigan Parks. *Journal of Community Health, 37*(2), 507-512. doi: 10.1007/s10900-011-9471-6.	Exclude – no change in the built environment	3
Robertson, L. B., Ward Thompson, C., Aspinall, P., Millington, C., McAdam, C., & Mutrie, N. (2012). The influence of the local neighbourhood environment on walking levels during the walking for wellbeing in the west pedometer-based community intervention. *Journal of Environmental and Public Health, 2012*(974786). doi: doi:10.1155/2012/974786.	Exclude – no built environment intervention and was a multi-component intervention	3
Rosenberg, D., Kerr, J., Sallis, J. F., Patrick, K., Moore, D. J., & King, A. (2009). Feasibility and outcomes of a multilevel place-based walking intervention for seniors: A pilot study. *Health & Place, 15*(1), 173-179.	Exclude – multi-component intervention	4
Sadler, R. C., Gilliland, J. A., & Arku, G. (2013). A Food Retail-Based Intervention on Food Security and Consumption. *International Journal of Environmental Research and Public Health, 10*(8), 3325-3346.	Exclude – not the same participants included at follow-up as at baseline	2
Semenza, J. C., March, T. L., & Bontempo, B. D. (2007). Community-initiated urban development: an ecological intervention. *Journal of Urban Health, 84*(1), 8-20.	Exclude – multi-component (residents were involved in the regeneration)	4
Tester, J., & Baker, R. (2009). Making the playfields even: evaluating the impact of an environmental intervention on park use and physical activity. *Prev Med, 48*(4), 316-320.	Exclude – not including local residents only as participants	2
Trayers, T., & Lawlor, D. A. (2005). The Jech Gallery: an environmental face lift: the Dings Home-Zone project. *J Epidemiol Community Health, 59*(10), 885.	Exclude – protocol/description paper	1
Tully, M. A., Hunter, R. F., McAneney, H., Cupples, M. E., Donnelly, M., Ellis, G., et al. (2013). Physical activity and the rejuvenation of Connswater (PARC study): protocol for a natural experiment investigating the impact of urban regeneration on public health. *BMC Public Health, 13*.	Exclude – protocol paper	1

van Lenthe, F. J., Martikainen, P., & Mackenbach, J. P. (2007). Neighbourhood inequalities in health and health-related behaviour: Results of selective migration? *Health & Place, 13*(1), 123-137.	Exclude – no actionable intervention	3
Vandermoere, F. (2008). Psychosocial health of residents exposed to soil pollution in a Flemish neighbourhood. *Social Science & Medicine, 66*(7), 1646-1657.	Exclude – no change in built environment	3
Veitch, J., Ball, K., Crawford, D., Abbott, G. R., & Salmon, J. (2012). Park Improvements and Park Activity A Natural Experiment. *American Journal of Preventive Medicine, 42*(6), 616-619.	Exclude - participants do not specifically include local residents (only park users)	2
*Veitch, J., Salmon, J., Carver, A., Crawford, D., Giles-Corti, B., & Timperio, A. (2014). REVAMP: A natural experiment to examine the impact of park renewal on park-use and park-based physical activity. *Journal of Science and Medicine in Sport, 18*, e146-e147. doi:http://dx.doi.org/10.1016/j.jsams.2014.11.154	Exclude – intercept and cross-sectional surveys (not the same participants at baseline and follow-up)	2
*Veitch, J., Salmon, J., Carver, A., Timperio, A., Crawford, D., Fletcher, E., & Giles-Corti, B. (2014). A natural experiment to examine the impact of park renewal on park-use and park-based physical activity in a disadvantaged neighbourhood: the REVAMP study methods. *BMC public health, 14*, 600. doi:http://dx.doi.org/10.1186/1471-2458-14-600	Exclude – protocol paper	1
Venegas-Sanchez, J., Rivadeneyra-Sicilia, A., Bolivar-Munoz, J., Lopez-Fernandez, L. A., Martin-Olmedo, P., Fernandez-Ajuria, A., . . . Artundo-Purroy, C. (2013). [Health impact assessment of the San Fernando street renewal project in Alcala de Guadaira (Seville, Spain)]. *Gac Sanit, 27*(3), 233-240. doi: 10.1016/j.gaceta.2012.08.002.	Exclude - Spanish	5
Ward-Thompson, C., Roe, J., & Aspinall, P. (2013). Woodland improvements in deprived urban communities: What impact do they have on people's activities and quality of life? *Landscape and Urban Planning, 118*, 79-89.	Exclude – not the same participants at follow-up as included in the sample at baseline	2
Webb, Elizabeth, Blane, David, McMunn, Anne, & Netuveli, Gopalakrishnan. (2011). Proximal predictors of change in quality of life at older ages. *Journal of Epidemiology & Community Health, 65*(6), 542-547. doi: 10.1136/jech.2009.101758.	Exclude – no change in the built environment	3

Werngren-Elgstrom, M., Carlsson, G., & Iwarsson, S. (2009). A 10-year follow-up study on subjective well-being and relationships to person-environment (P-E) fit and activity of daily living (ADL) dependence of older Swedish adults. *Archives of Gerontology and Geriatrics, 49*(1), E16-E22.	Exclude – no change in the built environment	3
Wickrama, T., Merten, M. J., & Wickrama, K. A. S. (2012). Early community influence on young adult physical health: Race/ethnicity and gender differences. *Advances in Life Course Research, 17*(1), 25-33.	Exclude – no change in the built environment	3
Wolf-Ostermann, K., Naber, S., & Graske, J. (2014). Housing Project 'Living Independently with Intensive Support (WmI)': The Scientific Evaluation. *Rehabilitation, 53*, S4-S43.	Exclude – not available in Australia	5
Wu, X. G., Tang, Z., Fang, X. H., Liu, H. J., Diao, L. J., & Xiang, M. J. (2004). [Evaluation of predictive effect of some health-related indices on deaths among ageing residents through a 8-years' follow-up study in Beijing]. *Zhonghua Liu Xing Bing Xue Za Zhi, 25*(4), 325-328.	Exclude – no change in the built environment	3
Xu, H., Short, S. E., & Liu, T. (2013). Dynamic relations between fast-food restaurant and body weight status: a longitudinal and multilevel analysis of Chinese adults. *Journal of Epidemiology & Community Health, 67*(3), 271-279.	Exclude - observational	1
Yan, X. Y. W., & England, M. E. (2001). Design evaluation of an Arctic research station - From a user perspective. *Environment and Behavior, 33*(3), 449-470.	Exclude – not residential neighbourhood (work)	3
Yassi, A., Fernandez, N., Fernandez, A., Bonet, M., Tate, R. B., & Spiegel, J. (2003). Community participation in a multisectoral intervention to address health determinants in an inner-city community in central Havana. *J Urban Health, 80*(1), 61-80. doi: 10.1093/jurban/jtg061.	Exclude – multi-component intervention	4
Yiannakoulias, N., Scott, D. M., Rowe, B. H., & Voaklander, D. C. (2011). Child pedestrian injuries and urban change. *Injury Prevention, 17*(1), 9-14.	Exclude – not the same participants at follow-up as at baseline	2
Yzermans, C. J., Donker, G. A., Kerssens, J. J., Dirkzwager, A. J., Soeteman, R. J., & ten Veen, P. M. (2005). Health problems of victims before and after disaster: a longitudinal study in general practice. *International Journal of Epidemiology, 34*(4), 820-826.	Exclude – natural disaster not a built environment intervention	3

Yu, Q., Scribner, R., Carlin, B., Theall, K., Simonsen, N., Ghosh-Dastidar, B., et al. (2008). Multilevel spatio-temporal dual change point models for relating alcohol outlet destruction and changes in neighbourhood rates of assaultive violence. *Geospat Health, 2*(2), 161-172.	Exclude – violence rate was the outcome, not a health measure	6
Zhang, S. C., Liu, X., Necheles, J., Tsai, H. J., Wang, G. Y., Wang, B. Y., . . . Wang, X. B. (2010). Genetic and Environmental Influences on Serum Lipid Tracking: A Population-Based, Longitudinal Chinese Twin Study. *Pediatric Research, 68*(4), 316-322.	Exclude - observational	1
Zimmer, Z., & Korinek, K. (2010). Shifting coresidence near the end of life: comparing decedents and survivors of a follow-up study in China. *Demography, 47*(3), 537-554.	Exclude – not a built environment intervention	3
Ziviani, J., Macdonald, D., Ward, H., Jenkins, D., & Rodger, S. (2008). Physical activity of young children: a two-year follow-up. *Physical & Occupational Therapy in Pediatrics, 28*(1), 25-39.	Exclude – not a built environment intervention	3
